# Evolution of East Asia’s Arcto-Tertiary relict *Euptelea* (Eupteleaceae) shaped by Late Neogene vicariance and Quaternary climate change

**DOI:** 10.1186/s12862-016-0636-x

**Published:** 2016-03-22

**Authors:** Ya-Nan Cao, Hans Peter Comes, Shota Sakaguchi, Lu-Yao Chen, Ying-Xiong Qiu

**Affiliations:** Key Laboratory of Conservation Biology for Endangered Wildlife of the Ministry of Education, and College of Life Sciences, Zhejiang University, Hangzhou, 310058 China; Department of Ecology & Evolution, Salzburg University, A-5020 Salzburg, Austria; Laboratory of Plant Evolution and Biodiversity, Graduate School of Arts and Sciences, The University of Tokyo, Tokyo, 153-0041 Japan

## Abstract

**Background:**

The evolutionary origin and historical demography of extant Arcto-Tertiary forest species in East Asia is still poorly understood. Here, we reconstructed the evolutionary and population demographic history of the two extant *Euptelea* species in China (*E. pleiosperma*) and Japan (*E. polyandra*)*.* Chloroplast/nuclear DNA sequences and microsatellite loci were obtained from 36 *Euptelea* populations to explore molecular structure and diversity in relation to past and present distributions based on ecological niche modelling (ENM). Time-calibrated phylogenetic/phylogeographic inferences and niche-identity tests were used to infer the historical process of lineage formation.

**Results:**

*Euptelea pleiosperma* diverged from *E. polyandra* around the Late Miocene and experienced significant ecological differentiation. A near-simultaneous diversification of six phylogroups occurred during the mid-to-late Pliocene, in response to the abrupt uplift of the eastern Tibetan Plateau and an increasingly cooler and drier climate. Populations of *E. pleiosperma* seem to have been mostly stationary through the last glacial cycles, while those of *E. polyandra* reflect more recent climate-induced cycles of range contraction and expansion.

**Conclusions:**

Our results illustrate how Late Neogene climatic/tectonic changes promoted speciation and lineage diversification in East Asia’s Tertiary relict flora. They also demonstrate for the first time a greater variation in such species’ responses to glacial cycles in Japan when compared to congeners in China.

**Electronic supplementary material:**

The online version of this article (doi:10.1186/s12862-016-0636-x) contains supplementary material, which is available to authorized users.

## Background

Because of the lack of extensive glaciations there during the Quaternary [1], the warm temperate climate zones of East Asia are considered to be one of the most important areas for the long-term persistence of Arcto-Tertiary forest flora [2]. In fact, although there are *c.* 600 genera of the Arcto-Tertiary relict flora endemic to East Asia (e.g. *Cercidiphyllum*, *Davidia*, *Euptelea*, *Ginkgo biloba*, *Tetracentron*), their evolution has been less well elucidated [3, 4]. Our limited understanding of the evolution of these Arcto-Tertiary plants is partly attributable to the scarce vascular plant fossils in the Tertiary and Quaternary beds of East Asia [5]. In addition, Arcto-Tertiary relict species have persisted with little morphological change [6], which might mask more recent diversification events. Finally, the modern patterns of endemism and disjunction of these relicts (e.g. between China and Japan) provide only limited insight into how past changes in climate, tectonics, and/or sea-level influenced their range dynamics and population/species divergence [7–10].

Nonetheless, recent molecular phylogeographic studies suggest that most Arcto-Tertiary relicts endemic to China have been little affected by Quaternary glacial-interglacial alternation [4, 11, 12]. In those instances, species likely tracked their climatic niche boundaries locally through altitudinal migration in a topographically complex landscape [13–15]. As a possible exception, the Chinese-Japanese disjunct *Cercidiphyllum japonicum* responded to Quaternary climate change through latitudinal range shifts, in both regions, which likely reflects the species’ high(er) pollen/seed dispersal capacity, sprouting ability, and/or climatic (drought/cold) tolerance [3]. Hence, these different responses may be largely explained by taxon-specific differences in migratory capacity and/or eco-climatic niche preferences [13, 16, 17]. However, additional studies are needed to gain a better understanding of when Arcto-Tertiary relict plants in East Asia diversified and whether and how they have responded to Late Neogene/Quaternary environmental change in the extent and connectivity of their habitats.

*Euptelea* Sieb. et Zucc., is the only genus in Eupteleaceae, comprising two extant species, *E. pleiosperma* Hook. f. et Thoms. and *E. polyandra* Sieb. et Zucc. [18, 19] (Additional file 1: Figure S1a). Both are diploid (2*n* = 28) [20, 21], small- to medium-sized, broad-leaved deciduous trees with bisexual and wind-pollinated flowers that turn into winged fruitlets (‘samaras’), dispersed by gravity, wind and/or water [22]. *E. pleiosperma* has its main centre of distribution in regions bordering the eastern margin of the Qinghai-Tibetan Plateau (QTP)/Hengduan Mts. and southwest China (Sichuan Basin/Yungui Plateau), with scattered occurrences in central/east China and northeast India/Assam; its populations are distributed in isolated stands across a wide range of altitudes (*c*. 700–3600 m above sea level) in mountain riparian warm-temperate deciduous (hereafter ‘WTD’) forest [23]. By contrast, *E. polyandra* is restricted to the riparian WTD forest of south-central Japan (*c*. 100–1600 m above sea level) [24–27] (Additional file 1: Figure S1a).

*Euptelea* has extensive fossil records throughout the Northern Hemisphere, extending to at least the Palaeocene [28–32]. In East Asia, *Euptelea*-like fossils have been reported from the Mid-Miocene of southeast China [33] and from the Pliocene to the Mid-Pleistocene of central Japan [2, 34, 35] (Additional file 1: Figure S1b). Although *E. polyandra* had initially been classed as a member of Trochodendraceae [36], Smith [37] incorporated this species into the unigeneric family Eupteleaceae, consisting of only *E. pleiosperma* at that time. Previous phylogenetic analyses [38] supported *E. pleiosperma* and *E. polyandra* as separate species, but only two samples from each species were included. Moreover, *Euptelea* is part of the earliest-diverging lineage of Ranunculales (along with Circaeasteraceae, Papaveraceae, Lardizabalaceae, Menispermaceae, Berberidaceae, Ranunculaceae), which, in turn, appear at the base of eudicots [38–40]. However, no information is available on the divergence times between and within the two species, to say nothing about their genetic architecture, demographic history, and ecological (climatic) niche divergence. Notably, this latter aspect has rarely been examined in phylogeographic studies of East Asia’s temperate flora in general, and its Tertiary relict components in particular [3].

Here, we use an integrative approach by combining phylogeographic and fossil-calibrated phylogenetic analyses with (palaeo)climatic data and niche identity tests to clarify the time and mode of lineage divergence and historical demography of *Euptelea*. Our specific aims were to (i) reveal the genetic relationships of the two extant species (e.g. are they recognizable as separate genetic entities?), and approximate divergence times of interspecific and intraspecific lineages; (ii) determine climatic niche divergence of both species and the two major lineages identified in *E. pleiosperma*; and (iii) illustrate the genetic diversity and population demographic patterns of both species.

## Methods

### Population sampling

Leaf material was obtained from 26 populations (*n* = 350) of *E. pleiosperma* and 10 populations (*n* = 90) of *E. polyandra* (Additional file 2: Table S1, Additional file 1: Figure S1a), covering most of the geographical range of the genus in East Asia except northeast India/Assam [37]. Fresh leaves were collected from each individual and dried with silica gel.

### DNA isolation, sequencing and microsatellite genotyping

Total genomic DNA was extracted from the silica-dried leaf tissue using DNA Plantzol (Invitrogen) following the manufacturer’s protocol. For the phylogeographic DNA surveys, two intergenic spacer (IGS) regions (*psb*A–*trn*H, *rpo*B–*trn*C) and the *rpL16* intron of chloroplast (cp) DNA, revealing high levels of variation in a preliminary screen, and the entire internal transcribed spacer (ITS) region of nuclear ribosomal (nr) DNA were selected for sequencing. Subsets of the samples were sequenced at cpDNA regions (*n* = 204 and 82 for *E. pleiosperma* and *E. polyandra*, respectively; Additional file 2: Table S1) and ITS region (*n* = 113 and 48 for *E. pleiosperma* and *E. polyandra*, respectively; Additional file 2: Table S1). A subset of individuals was also sequenced at three additional cpDNA regions (*pet*N–*trn*C, *mat*K, *rbc*L) for phylogenetic analysis (35 individuals, representing 35 chlorotypes; see the Results section). The primers and methodology for PCR amplification of these cpDNA and ITS regions were described elsewhere [41–44]. The PCR products were directly sequenced in both directions with an ABI 3730XL DNA Analyzer (Applied Biosystems, Foster City, CA, USA). The raw sequence data were adjusted, assembled and aligned in geneious v.4.8.5 [45]. All cpDNA and ITS haplotype sequences have been deposited in GenBank (see Additional file 3: Table S2 for accession numbers).

Following Zhang et al. [46] and Wu et al. [47], eight nSSR markers (EP04, EP06, EP10, EP59, EP87, EP91, EP278, EP294) and amplification protocols, specially developed for *Euptelea*, were used for genotyping all samples (*n* = 440). The PCR products were also loaded on an ABI 3730XL DNA Analyzer, and the data were scored and compiled using genemarker v.2.2.0 (SoftGenetics, State College, PA, USA).

### Phylogeographic analyses of cpDNA, ITS, and nSSRs

For both cpDNA and ITS, all indels and inversions were treated as single mutation events, and coded as substitutions with equal weight [48]. Haplotype (*h*) and nucleotide (*π*) diversities [49] were calculated for each population (*h*_S_*, π*_S_) and species (*h*_T_*, π*_T_) using dnasp v.5.1 [50]. tcs v.1.21 [51] was used to estimate an unrooted network of *Euptelea* haplotypes that illustrates all linkages with a > 95 % probability of being most parsimonious. Analysis of molecular variance (AMOVA) in arlequin v.3.1 [52] was performed using *Φ*-statistics to quantify the composition of total genetic (cpDNA, ITS) variance, with significance of fixation indices tested using 10,000 permutations [53]. Population differentiation for ordered (*N*_ST_) and unordered (*G*_ST_) haplotypes (cpDNA) were obtained with permut [54, 55] to test whether *N*_ST_ is significantly larger than *G*_ST_ (1000 permutations), indicating the presence of phylogeographic structure.

For the nSSR dataset, one *E. pleiosperma* and two *E. polyandra* populations with small sample sizes (*n* < 8) were removed from all population-level analyses of genetic diversity and differentiation, limiting those analyses to 33 populations (marked with an asterisk in Additional file 2: Table S1). All eight loci were checked for frequencies of null alleles using the Expectation Maximization (EM) algorithm [56] implemented in freena [57]. We measured unbiased genetic diversity [58, 59] across all loci for all populations with *n* ≥ 8, and calculated their allelic (*A*_R_) and private allelic (*PA*_R_) richness (standardized for 8 individuals using rarefaction) in hp-rare v.1.1 [59]. fstat v.2.9.3 [60] was used to estimate the total number of detected alleles (*N*_A_), gene diversity (*H*_S_), and genetic differentiation (*F*_ST_) [61] between populations (*n* ≥ 8) per-locus and overall.

Bayesian clustering of individuals was performed in structure v.2.3.4 [62, 63] for the entire nSSR dataset (i.e. 36 populations) and each species. We used the admixture model without prior information on population membership, and assumed independent allele frequencies among populations. The number of clusters (*K*) was set to vary depending on the data set. For each value of *K*, we conducted 10 independent simulations with both a burn-in and run length of 100,000 Markov chain Monte Carlo (MCMC) replications. The number of gene pools (i.e. the optimal *K*) was inferred by estimating lnP(*D*) [64] and *ΔK* [65]. AMOVAs were carried out in arlequin with *R*-statistics as described above.

### Phylogenetic divergence time estimation and demographic inference

To relate the diversification of major cpDNA lineages of *Euptelea* to Neogene events, we adopted a two-step approach for estimating divergence times in beast v.1.7.5 [66] by taking advantage of calibration points used in previous studies of Ranunculales [38, 67–69]. Taxon sampling has been shown to affect dates among basal Eudicots; increased taxon sampling, particularly in underrepresented clades, will likely improve the accuracy of divergence time estimation [70]. In this study, 33 Ranunculales species, including 13 species of Ranunculaceae, 13 of Berberidaceae and 5 of Menispermaceae (core Ranunculales), and the two species of Eupteleaceae (basal Ranunculales), plus representatives of Sabiaceae (*Meliosma*, *Sabia*) and Proteales (*Nelumbo*, *Platanus*) of basal eudicots used as outgroups (Additional file 3: Table S2) [38], were selected for estimating the crown group age of *Euptelea*. These selected species thus represent all major lineages of Ranunculales based on more broadly sampled earlier phylogenetic studies [38]. First, we retrieved sequences of two cpDNA regions (*rbc*L, *mat*K) and 26S nrDNA from GenBank for the 33 Ranunculales species. Sequences were assembled together and then Bayesian searches for tree topologies and node ages of this cpDNA + 26S nrDNA dataset were implemented in beast with a GTR + G + I substitution model selected by jmodeltest v.2.1.4 [71], and an uncorrelated lognormal relaxed clock [72]. A Yule process was chosen as tree prior. Four calibration points were used to determine minimum age constraints on specific node priors (see nodes 1–4 in Fig. 4a). The first calibration point is based on the oldest confirmed fruit fossil of Sabiaceae from the Cretaceous in Czech Republic [73], which is unambiguously assigned to *Insitiocarpus moravicus*. Following Anderson et al. [67], we assigned this fruit fossil to the stem of Sabiaceae (98 Ma; see node 1 in Fig. 4a). The flower fossil of *Platanocarpus brookensis* from the Mid-Cretaceous in North America [74] was taken as the second calibration point and was used to calibrate the crown age of *Nelumbo* and *Platanus* (98 Ma; see node 2 in Fig. 4a) [69]. Following Anderson et al. [67], we used the endocarp fossil of *Prototinomiscium* (Menispermaceae) from the Cretaceous of central Europe [73, 75] to calibrate the crown age of the Menispermeaceae-Berberidaceae-Ranunculaceae clade (91 Ma; see node 3 in Fig. 4a). Finally, following Wang et al. [68], we used a leaf fossil of *Mahonia* from the Mid-Eocene in North America [5] to calibrate the crown age of the *Ranzania*-*Mahonia*-*Berberis* clade (45 Ma; see node 4 in Fig. 4a). All calibration points were modeled using a lognormal prior with a zero offset 98, 98, 91 and 45 Ma, respectively; by default, lognormal mean of 0 and lognormal SD of 0.5 were used to constrain each node (see nodes 1–4 in Fig. 4a). To evaluate the robustness of the resulting age estimates, we removed each calibration sequentially while holding the rest three calibrations [76].

**Fig. 1:**
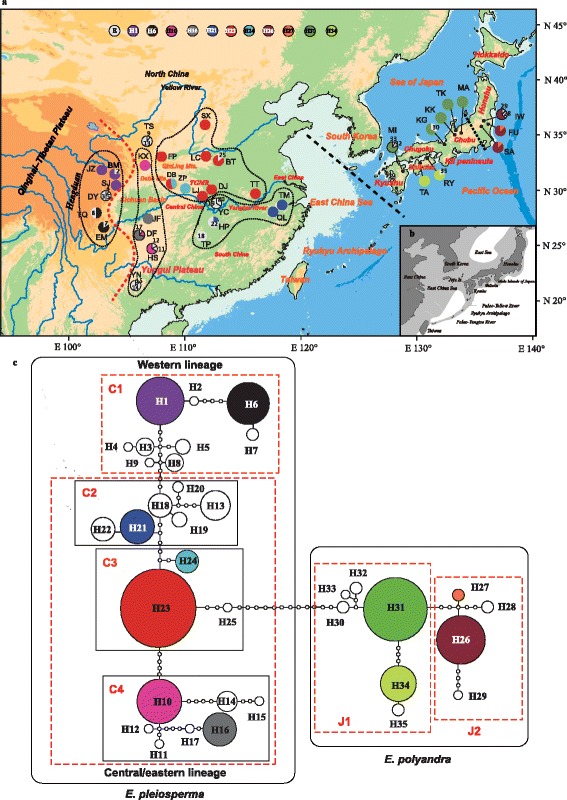
**a** Distribution of chlorotypes in 26 populations of *E. pleiosperma* (China) and 10 populations of *E. polyandra* (Japan) (see Additional file 2: Table S1 for population codes). Chlorotypes shared among populations are denoted by colour, while population-specific ones (‘E’) are white. The red and black dashed lines represent, respectively, the Sino-Himalayan/Sino-Japanese Forest boundary and the species’ boundary across the East China Sea region. The black dotted lines delimitate the four phylogroups of *E. pleiosperma* (i.e. population groups that share closely related chlorotypes). The black dotted line in Japan marks the boundary between ‘southern’ and ‘central’ phylogroups in *E. polyandra*. Abbreviations: TGMR, Three Gorges Mountain Region. **b** Dark-shaded areas in the inset indicate current mainland and island configurations, and light-shaded areas indicate exposed coastal areas and sea basins of East Asia during Late Pleistocene sea-level alterations owing to glaciations (modified after Park) [125]. **c** tcs-derived network of genealogical relationships between the 35 chlorotypes of the two species. Each circle denotes a single chlorotype with size proportional to frequency. Small open circles represent missing chlorotypes. The four clades (phylogroups) of *E. pleiosperma* identified by tcs were denoted as C1, C2, C3, C4, while those two clades (phylogroups) of *E. polyandra* were represented by J1 and J2. The baseline map was created by us using ArcGIS v.10.2.2

In a second step, we applied a similar beast analysis to all 35 cpDNA (*psb*A–*trn*H, *rpo*B–*trn*C, *rpL16* intron, *pet*N–*trn*C, *mat*K, *rbc*L) haplotype sequences of *Euptelea* identified in the phylogeographic survey (see the Results section; Fig. 1) to determine intrageneric node ages, with *Platanus occidentalis* used as outgroup (Fig. 4b). We employed the same settings as in the first step, but for a GTR + G substitution model and a constant-size coalescent tree prior. To calibrate the root node (see node B in Fig. 4b), we used the median crown age of *Euptelea* (5.46 Ma) estimated from the Ranunculales phylogeny (see node A in Fig. 4a) with a normal distribution (mean, 5.46 Ma; SD, 2.5 Ma; 95 % confidence interval [CI], 1.35–9.58 Ma). For both beast analyses, MCMC runs were conducted, each of 2 × 10^7^ generations, with trees sampled every 2000 generations, after the initial 10 % were discarded as burn-in. The MCMC samples were examined in tracer v.1.5 [77] to confirm sampling adequacy and convergence of the chains to a stationary distribution.

For each species, and major (cpDNA) phylogroups (see the Results section), we constructed Bayesian skyline plots (BSPs) in beast to date changes in effective population size (*N*_e_) over time [78]. We assumed an uncorrelated lognormal relaxed clock with substitution rates obtained from the intrageneric beast analyses described earlier. The MCMCs were run for 1 × 10^7^ generations with trees sampled every 1000 generations, following a burn-in of the initial 10 % cycles. Three replicate runs were performed to confirm convergence.

### Ecological niche modelling and niche identity tests

ENMs were generated using the maximum entropy method implemented in maxent v.3.2.1 [79] to predict the potentially suitable climate envelopes for each *Euptelea* species in the present, and in two past periods: the Last Glacial Maximum (LGM), *c.* 21 kya; and the Last Interglacial (LIG), *c.* 130 kya. Information on the geographic distribution of *Euptelea* was based on a total of 470 presence records, including 99 for *E. pleiosperma* [i.e. 26 sample sites of this study plus 73 records from the Chinese Virtual Herbarium (http://www.cvh.org.cn)] and 371 for *E. polyandra* [i.e. 10 sample sites of this study and 361 records from the S-Net data portal (http://science-net.kahaku.go.jp/)], with the removal of duplicate records within each pixel (2.5 arc-min; *c*. 5 km). We compiled 19 environmental variables for each period from the WORLDCLIM database with a resolution of 2.5 arc minutes [80] for each environment layer (Additional file 2: Table S1). Based on 99 records for *E. pleiosperma* and 371 records for *E. polyandra*, a current distribution model was developed for each species using five bioclimatic data layers (annual mean temperature, annual precipitation, temperature annual range, mean temperature of wettest quarter, and precipitation seasonality) at 2.5 arc-minutes resolution for the present (1950–2000). This limited bioclimatic dataset avoided containing highly correlated variables (data not shown), and therefore prevented potential overfitting [81]. By adopting default settings with 10 replicates, we used 75 % of species records to train the model and 25 % to test the model. The area under the ROC curve (AUC) was calculated for each run to assess the model accuracy. Values between 0.7 and 0.9 indicate good discrimination [82]. We then projected the established model onto the reconstructed climatic conditions for the LGM and LIG periods, as simulated by the community climate system model v.3.0 [83], provided by the Palaeoclimate Modelling Intercomparison Project (http://pmip2.lsce.ipsl.fr/). Following Sakaguchi et al. [84], the present-day and LGM palaeoclimate layers were prepared in 2.5 arc-minutes resolution. To account for changes in palaeo-coastlines in the East China Sea (ECS) region during the LGM (*c*. -130 m lower than at present) [85], we used the seafloor topography data (ETOPO1) from the National Geophysical Data Center of National Oceanic and Atmospheric Administration (NOAA, Washington, DC, USA).

To examine potential environmental factors associated with the divergence between *E. pleiosperma* and *E. polyandra* (and between two major cpDNA lineages of the former species; see Results section), the means and standard errors of those 19 bioclimatic variables were then calculated for each species (lineage) and compared to detect significant differences using two-tailed *t*-tests in R v.3.1.1 [86]. We also performed niche identity tests [87] in enmtools v.1.4.3 [88] with 500 pseudoreplicates based on (i) the five bioclimatic variables used for ENM (see above); and (ii) all the 19 BIOCLIM variables together, for testing the null hypothesis that the two species (lineages) are occupying identical climatic environments (‘niches’). To this aim, we compared their actual niches to a distribution of niche similarities obtained from pairs of pseudoniches based on randomly sampled occurrence points [89]. Niche overlap was quantified by both Schoener’s *D* [90] and the standardized Hellinger distance (*I*).

## Results

### CpDNA phylogeography and diversity

The total alignment of three cpDNA regions sequenced across the 286 individuals of *Euptelea* was 2078 bp in length, including 25 single-nucleotide variable sites, five indels (5–18 bp), and one inversion (23 bp; Additional file 4: Table S3). Together, these 31 polymorphisms identified 35 haplotypes (‘chlorotypes’, H1–35), of which 25 were specific to *E. pleiosperma* (H1–25) and 10 to *E. polyandra* (H26–35; Fig. 1a). Of the 35 chlorotypes identified in *Euptelea*, 24 were population-specific and nearly half of the populations of each species were fixed for a single chlorotype (Fig. 1a, Additional file 2: Table S1). Accordingly, non-hierarchical AMOVA (Table 1) revealed that most of the cpDNA sequence variation within each species resided among populations (*E. pleiosperma/E. polyandra*: *Φ*_ST_ = 0.87/0.75; both *P* < 0.01), and this variation exhibited significant phylogeographic structure (*E. pleiosperma/E. polyandra*: *N*_ST_ = 0.847/0.879 > *G*_ST_ = 0.835/0.836; both *P* < 0.05). In the tcs network (Fig. 1b), these species-specific chlorotypes formed two clades separated by thirteen mutational steps (between H25 and H30). This pronounced subdivision was also indicated by hierarchical AMOVA with 72.52 % among-species variation (Table 1).

**Table 1:** The analysis of molecular variance (AMOVA) for cpDNA data and nuclear (nSSRs and ITS) data for *Euptelea* and its two species

	cpDNA			ITS (nrDNA)			nSSRs		
Source of variation	d.f.	Percentage of total variance (%)	*Φ*-statistics	d.f.	Percentage of total variance (%)	*Φ*-statistics	d.f.	Percentage of total variance (%)	*R*-statistics
*Euptelea*									
Among species	1	72.52	*Φ*_CT_ = 0.73	1	80.08	*Φ*_CT_ = 0.81	1	22.94	*R*_CT_ = 0.23
Among populations within species	34	23.68	*Φ*_SC_ = 0.86	34	6.59	*Φ*_SC_ = 0.33	34	12.16	*R*_SC_ = 0.16
Within populations	250	3.8	*Φ*_ST_ = 0.96	125	13.33	*Φ*_ST_ = 0.87	844	64.9	*R*_ST_ = 0.35
*E. pleiosperma*									
Among populations	25	87.31	*Φ*_ST_ = 0.87	25	36.39	*Φ*_ST_ = 0.36	25	16.71	*R*_ST_ = 0.17
Within populations	178	12.69		87	63.61		674	83.29	
*E. polyandra*									
Among populations	9	74.9	*Φ*_ST_ = 0.75	9	31.24	*Φ*_ST_ = 0.31	9	10.44	*R*_ST_ = 0.10
Within populations	72	25.1		38	68.76		170	89.56	

Among the 25 chlorotypes of *E. pleiosperma*, eight (H1–8) were specific to a group of six populations (JZ, BM, SJ, DY, TQ, EM) from the region bordering the eastern QTP/Hengduan Mts. (hereafter ‘C1’; see dotted lines in Fig. 1a). By contrast, the remaining chlorotypes were largely specific to three ‘central-eastern’ regions: (i) the northern and, in particular, southern flanks of the Sichuan Basin, including the Yungui Plateau (‘C4’: H10–11, H14–17); (ii) the mid-lower reaches of the Yangtze (‘C2’: H18–22); and (iii) areas north of this river (‘C3’: H23–25). The parsimony network (Fig. 1b) grouped these chlorotypes into two lineages (western vs central/eastern) and four distinct phylogroups (i.e. C1–C4) according to geography; the only exceptions were H9 and H13, which resided in a single population (TS) from the Sichuan Basin (hereafter ‘SB’) region (Fig. 1a and b; Additional file 2: Table S1), however, were assigned to two distinct phylogroups (C1 and C2, respectively). In *E. polyandra*, the two distinct phylogroups (J1 vs J2) identified segregated populations into the southern and central parts of Japan (i.e. Kyushu, Shikoku, south Honshu/Chubu vs north Honshu; see Fig. 1a, b).

### ITS phylogeography and diversity

**Fig. 2:**
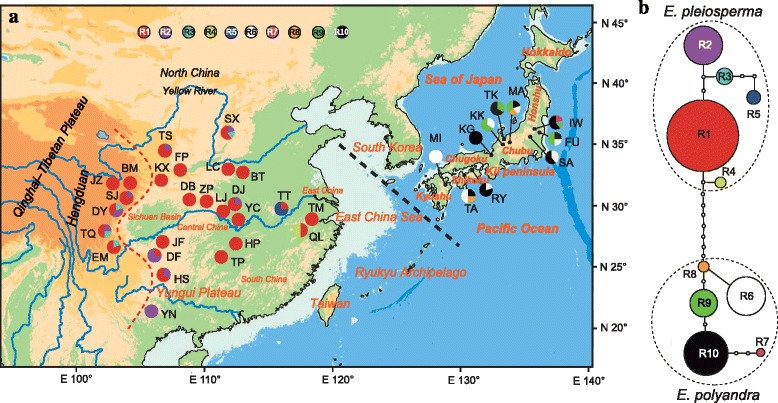
**a** Distribution of ITS ribotypes in the 36 populations of *Euptelea* (see Additional file 2: Table S1 for population codes). Red and black dashed lines are identified in Fig. 1. **b** tcs-derived network of genealogical relationships between the 10 ribotypes of the two species. Each circle denotes a single ribotype, with size proportional to frequency. Small open circles represent missing ribotypes

The total alignment of the ITS region sequenced across the 161 individuals of *Euptelea* was 691 bp in length, including 18 single-nucleotide variable sites. These polymorphisms yielded 10 ribotypes (R1–10; Additional file 5: Table S4), with five each specific to *E. pleiosperma* (R1 − 5) and *E. polyandra* (R6–10). The parsimony network (Fig. 2) grouped *E. pleiosperma* vs *E. polyandra* ribotypes into distinct clades (‘lineages’), separated by nine mutational steps, resulting in 80.08 % among-species variation (*Φ*_CT_ = 0.80, *P* < 0.01; Table 1). In *E. pleiosperma*, the most basal ribotype (R1) was also the most frequent and widespread one, being fixed in 14 (out of 26) populations, while in *E. polyandra* almost all populations (8/10) were polymorphic. In contrast to cpDNA, most of the within-species ITS variation resided within populations (*E. pleiosperma*/*E. polyandra*: *Φ*_ST_ = 0.36/0.31, both *P* < 0.01; Table 1).

### Species subdivision and population genetic structure at nSSR loci

**Fig. 3:**
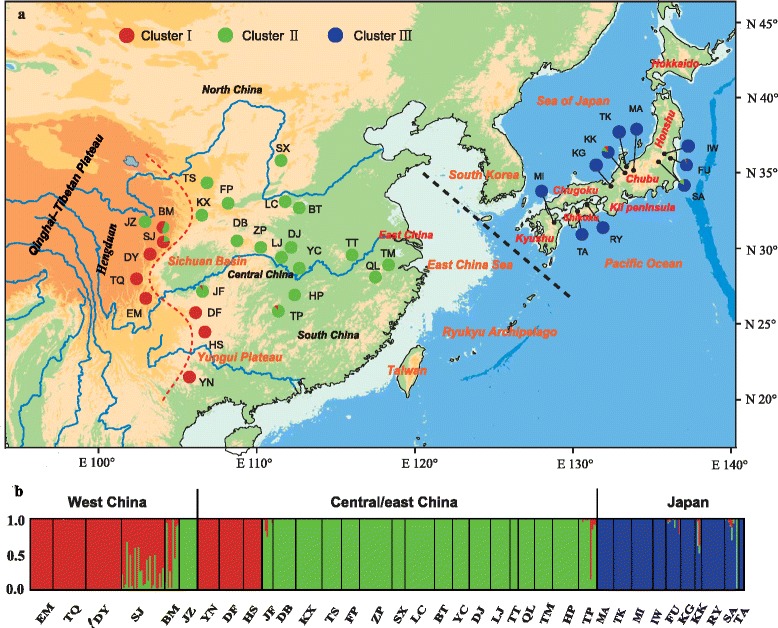
**a** Colour-coded grouping of the 36 *Euptelea* populations according to the three structure clusters (I–III) (see Additional file 2: Table S1 for population codes). Red and black dashed lines are identified in Fig. 1. **b** Histogram of the structure analysis for the model with *K* = 3 (according to both lnP(*D*) and *ΔK*; see Additional file 7: Figure S2). Each vertical bar represents one individual. The assignment ratio of each individual into one of the three clusters is shown along the y-axis. Each cluster is represented by a distinct colour

Across the 440 individuals of *Euptelea* genotyped at eight nSSR loci*,* we obtained altogether 150 alleles, ranging from 7 to 29 alleles per locus (for details see Additional file 6: Table S5). For the entire dataset, the *ΔK* statistic detected a rate change in lnP(*D*) corresponding to *K* = 3 (Additional file 7: Figure S2). Accordingly, individuals of *E. pleiosperma* segregated into cluster I (Fig. 3a and b) that was present at high frequency in western populations (except JZ) but also fixed in three populations (DF, HS, YN) from the southern SB region, while the rest of the central-eastern individuals were largely assigned to cluster II. By contrast, individuals of *E. polyandra* almost exclusively formed a distinct cluster (Fig. 3a and b). A separate structure analysis of *E. pleiosperma* resulted in similar patterns, while for *E. polyandra* individuals were variously assigned to three clusters without geographical structure (data not shown).

Hierarchical AMOVA (Table 1) supported significant nSSR variation between the two species (*R*_CT_ = 0.23, *P* < 0.01). Within each species, most of this variation resided within populations, with somewhat stronger population subdivision in *E. pleiosperma* (*R*_ST_ = 0.17, *P* < 0.01) than in *E. polyandra* (*R*_ST_ = 0.10, *P* < 0.01).

### Molecular dating and demographic analyses

**Fig. 4:**
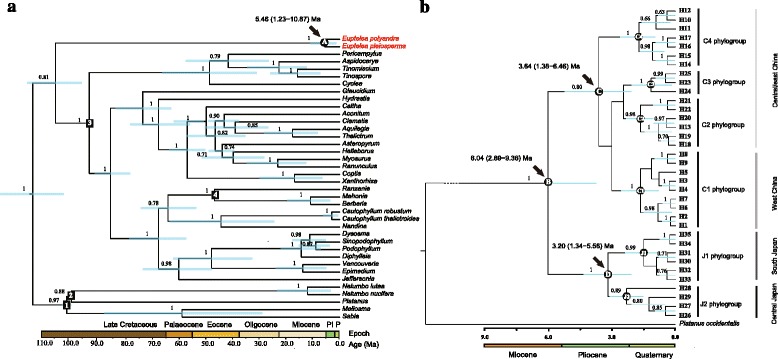
**a** beast-derived chronogram of Ranunculales based on cpDNA (*rbcL*, *matK*) + 26S nrDNA sequences with calibration points denoted by nodes 1–4 (see text and Table 2 for details); and **b** the crown age of *Euptelea* and its phylogroups based on cpDNA (*psb*A-*trn*H, *rpo*B-*trn*C, *rpL16*, *pet*N*-trn*C, *mat*K, *rbc*L) sequences. Blue bars on each node indicate 95 % highest posterior density (HPD) confidence intervals for time estimates (in million yr ago, Ma). Posterior probabilities (PP > 0.5) are shown above nodes. Mean divergence times and 95 % HPDs are summarized in Table 2. Chlorotypes are represented by letter codes (H1–35). Codes of subclades (phylogroups) are identified in Fig. 1

The beast-derived cpDNA + 26S nrDNA chronogram of Ranunculales (Fig. 4a) recovered *Euptelea* as sister to core Ranunculales (PP = 0.81) and estimated the crown age of *Euptelea* as *c.* 5.46 Ma [95 % highest posterior density (HPD): 1.23–10.87 Ma; see node A in Fig. 4a] (Table 2). Using this estimation as root prior for the cpDNA chronogram of *Euptelea* (Fig. 4b), the time to the most recent common ancestor (T_MRCA_) of all 35 chlorotypes was estimated as *c.* 6.04 Ma (95 % HPD: 2.89–9.36 Ma; see node B in Fig. 4b), suggesting a Late Miocene/Early Pliocene split between the two species. The T_MRCA_ for all chlorotypes of *E. pleiosperma* was estimated as *c*. 3.64 Ma (95 % HPD: 1.38–6.46 Ma; see node C in Fig. 4b), which in turn dates the species’ divergence into two lineages (see above) to around the Mid-Pliocene. The T_MRCA_ for all chlorotypes of *E. polyandra* (viz. its divergence into J1 vs J2 phylogroups) was estimated to be only slightly younger, *c.* 3.20 Ma (95 % HPD: 1.34–5.56 Ma; see node D in Fig. 4b). By contrast, the coalescence times of all six phylogroups of *Euptelea* most likely fall into the Quaternary [C1: 1.66 (0.47–3.31) Ma; C2: 1.66 (0.42–3.50) Ma; C3: 1.15 (0.17–2.68) Ma; C4: 1.73 (0.48–3.47) Ma; J1: 1.50 (0.36–3.07) Ma; J2: 2.31 (0.88–4.13); see nodes C1–4 and J1–2 in Fig. 4b and Table 2].

**Table 2:** Summary of cpDNA-based divergence time estimation results under a Bayesian approach using (a) a Yule speciation prior for the major clades of Ranunculales (see Fig. 4a) and (b) a coalescent prior for the crown group age of *Euptelea* and its phylogroups (Fig. 4b) as implemented in beast

Node	Type	Calibration age (95 % HPD)	Age	Reference
(a) Yule prior				
Node 1* (the stem age of Sabiaceae)	Fossil fruit	98.44–100.3	98	Knobloch & Mai (1986) [73]
Node 2* (the crown age of Proteales)	Fossil leaves	98.44–100.3	98	Crane et al. (1993) [74]
Node 3* (the stem age of Menispermeaceae)	Fossil fruit	91.44–93.28	91	Knobloch & Mai (1986) [73]
Node 4* (*Ranzania*/*Mahonia*)	Fossil leaves	45.44–47.28	45	Manchester (1999) [5]
Node A (crown age of *Euptelea*)		5.46 (1.23–10.87)		
(b) Coalescent prior				
Node B* (crown age of *Euptelea*)		6.04 (2.89–9.36)		
Node C (crown age of *E. pleiosperma*)		3.64 (1.38–6.46)		
Node C1 (crown age of C1 phylogroup)		1.66 (0.47–3.31)		
Node C2 (crown age of C2 phylogroup)		1.66 (0.42–3.50)		
Node C3 (crown age of C3 phylogroup)		1.15 (0.17–2.68)		
Node C4 (crown age of C4 phylogroup)		1.73 (0.48–3.47)		
Node D (crown age of *E. polyandra*)		3.20 (1.34–5.56)		
Node J1 (crown age of J1 phylogroup)		1.50 (0.36–3.07)		
Node J2 (crown age of J2 phylogroup)		2.31 (0.88–4.13)		

Estimates are given as mean ages (in millions of years) with 95 % HPD in parentheses. See methods for explanation of fossil calibration and model selection. Node numbers correspond to those in Fig. 4

^*^Calibration points

For *E. pleiosperma*, the Bayesian skyline plots analysis (Additional file 8: Figure S3a) showed that *N*_e_ kept steady growth up to the Late Pleistocene (*c.* 0.25 Ma), followed by stable population sizes until the present, while for each of the four phylogroups,*N*_e_ kept steady through time (data not shown). By contrast, for *E. polyandra* and its two phylogroups, this analysis consistently indicated stable population sizes up until the Late Pleistocene (*c.* 0.5 Ma), followed by a slight growth until the present (Additional file 8: Figure S3b–d).

### Ecological niche modelling and niche identity tests

**Fig. 5:**
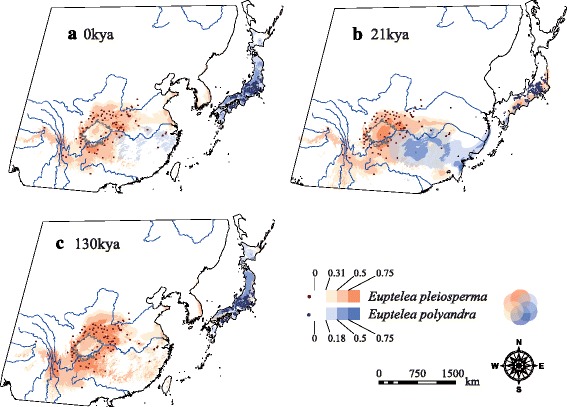
Potential distribution probability (in logistic value) of occurrence for *Euptelea* in East Asia. **a** At the present (0 kya); **b** at the Last Glacial Maximum (LGM: *c.* 21 kya) and **c** during the Last Interglacial (LIG: *c.* 130 kya). Presence records of *E. pleiosperma* in China (*n* = 99) and *E. polyandra* in Japan (*n* = 371) are plotted as purple and dark blue points in the maps, respectively. See text for details

The AUC values (± SD) for the distribution modelling, averaged over 50 replicates, were very high (0.958 ± 0.016 vs 0.971 ± 0.002 for *E. pleiosperma* and *E. polyandra*, respectively), indicating a good predictive model performance. The predicted distribution of *Euptelea* under current conditions (1950–2000) (Fig. 5a) was similar to its actual distribution, but there were also some predicted areas where either species does not occur at present, such as northern east China and south Korea (*E. pleiosperma*) or Hokkaido/north Honshu of Japan and central/east China (*E. polyandra*). During the LIG (*c.* 130 kya BP; Fig. 5c), the genus’ predicted distribution was more or less similar to that at present, but likely more favourable habitats especially for *E. pleiosperma* were in southwest China (Hengduan Mts.) and the surroundings of the Sichuan Basin. By contrast, at the LGM (*c.* 21 kya BP; Fig. 5b), the predicted suitable habitats of *E. pleiosperma* probably persisted around the latter basin, but also slightly expanded eastward, along the northern flanks of the middle-Yangtze River. At the same time, the predicted suitable habitats of *E. polyandra* became restricted to a narrow coastal strip along the Pacific Ocean side of southeast Japan. Notably, predicted suitable habitats likely existed for *E. pleiosperma* in the latter region as well, and conversely, for *E. polyandra* in central China (especially south of the middle-Yangtze River) and along the coasts of the exposed East China Sea continental shelf; however, for each species, hospitable habitats were apparently absent on the landbridge extending from east China across south Korea to south Japan (Fig. 5b).

**Fig. 6:**
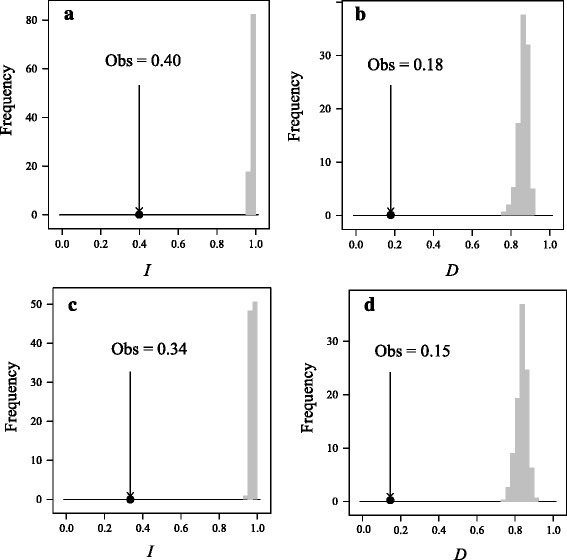
Niche identity test plots for five non-correlated (**a**, **b**) and nineteen (**c**, **d**) BIOCLIM variables between the two species of *Euptelea* as quantified by the standardized Hellinger distance (*I*) and Schoener’s *D* [91]. The vertical line in each plot represents the observed values of niche similarity (both *D* and *I*) while the histograms represent those of null distributions

**Fig. 7:**
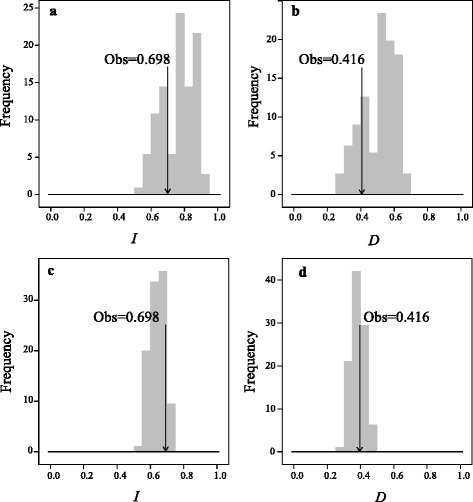
Results of the niche identity tests for five non-correlated (**a**, **b**) and nineteen (**c**, **d**) BIOCLIM variables between western and central-eastern populations of *E. pleiosperma*. The vertical line in each map represents the observed values of niche similarity (both *D* and *I*) while the histograms represent those of null distributions

Out of the 19 bioclimatic variables, all but three (bio2: mean diurnal temperature range; bio8: mean temperature of wettest quarter; bio10: mean temperature of warmest quarter), showed significant differences in mean values between *E. pleiosperma* and *E. polyandra* (Additional file 9: Figure S4). Randomization tests of niche identity (based on nineteen bioclimatic variables or five non-correlated variables) indicated that these sister species are not ecologically equivalent (*P* < 0.01) (Fig. 6), regardless of the measure of similarity used (Schoener’s *D* or Hellinger’s *I*). Within *E. pleiosperma*, seven (out of 19) bioclimatic variables showed significant (*P* < 0.05) differences between the western and central-eastern cpDNA lineages, i.e. in terms of isothermality (bio3), temperature/precipitation seasonality (bio4/bio15), and aspects of temperature (bio6, bio7, bio9, bio11; see Additional file 10: Figure S5). However, niche identity tests could not reject the null hypothesis that the two lineages occupy identical climatic niches, as *D* and *I* were non-significant (*P* > 0.05) in either instance (Fig. 7).

## Discussion

### Late Neogene speciation and diversification of *Euptelea*

Our phylogenetic and phylogeographic analyses based on cytoplasmic and nuclear (ITS, nSSR) data (Figs. 1, 2, 3 and 4) clearly support the monophyly of the genus *Euptelea* and provide the first evidence for the genetic distinctiveness of *E. pleiosperma* (China) and *E. polyandra* (Japan). In addition, although these ecological differences (Additional file 9: Figure S4; Fig. 6) could reflect evolutionary divergence of both species, they might simply reflect the fact that populations of both species are largely allopatric and thus are exposed to different environmental backgrounds. Hence, these species are diagnosable by geographical, morphological, molecular and ecological criteria.

Based on our cpDNA chronogram (Fig. 4b), this speciation event occurred *c.* 6.04 (95 % HPD, 2.89–9.36) Ma. Notably, this point estimate broadly coincides with an early stage of East China Sea seafloor exposure between the Eurasian mainland and the Japanese/Ryukyu Islands (7.0–5.0 Ma) [91, 92]. Hence, this landbridge could have allowed a common ancestor to expand its range from China to Japan, followed by range fragmentation either due to an increasingly cooler and drier climate around that time in Asia (*c.* 7–5.3 Ma) [4, 93, 94] and/or a subsequent rise in sea level. Of course, this vicariant scenario has to be treated with caution because of broad confidence limits around the above point estimate (2.89–9.36 Ma; Fig. 4b). Nonetheless, our data clearly indicate a long period of independent evolution of the two species, which ensured almost complete lineage sorting at chloroplast and nuclear loci. In support of this, our ENM for the LGM (and possibly earlier, colder periods) predicted climatically suitable areas in central/east China for *E. polyandra* and in southeast Japan for *E. pleiosperma*, but no such areas were predicted for either species on the ECS landbridge (Fig. 5b). Therefore, the East China Sea landbridge likely acted as a formidable barrier to the dispersal of *Euptelea* species during the LGM and earlier cold periods, despite its repeated exposure during the Quaternary (2.0–1.3 Ma, and 0.2–0.015 Ma) [91, 92, 95].

There is increasing evidence to suggest that the East China Sea landbridge acted as a ‘filter’ for East Asia’s temperate flora in selectively preventing or facilitating the dispersal of certain plant species [10, 96]. For instance, genetic breaks across the East China Sea have been observed in several understorey herbs or shrubs of WTD forest (e.g. *Kirengeshoma palmata*; *Platycrater arguta*; *Ligularia hodgsonii*) [89, 96–98]. However, in sharp contrast, no genetic breaks associated with the East China Sea were found in two widespread WTD forest tree species, namely *Cercidiphyllum japonicum* [3] and *Kalopanax septemlobus* [85]. As with *Euptelea* spp., *C. japonicum* is also an Arcto-Tertiary relict of riparian plant communities with similar reproductive characteristics (e.g. wind-dispersed pollen and seeds, vegetative propagation) [22, 99, 100]. However, *C. japonicum,* which is presently found up to north Japan, is relatively tolerant to cold and arid climates, which might have facilitated gene exchange across the glacially exposed ECS landbridge up until its latest submergence (*<*16 000 year bp; Fig. 5c) [3]. By contrast, *Euptelea* is less tolerant to cold, more drought-sensitive, and also much weaker in terms of sprouting ability [26, 100, 101]. We therefore conclude that the East China Sea landbridge provided a far less suitable (albeit unknown) environment for *Euptelea* relative to *Cercidiphyllum* due to taxon-specific differences in climate-related niche requirements and/or other biological (e.g. recruitment) properties [96].

Our molecular dating analyses (see Fig. 4b; Table 2) indicate that the onset of diversification in *E. pleiosperma* (node C, *c.* 3.64, 95 % HPD, 1.38–6.46 Ma) and *E. polyandra* (node D, *c.* 3.20, 95 % HPD, 1.34–5.56 Ma) occurred at the mid-to-late Pliocene. These broad HPD ranges preclude us from explaining any of these splits in terms of particular geological or palaeoclimatic events. Despite these caveats, we propose that our dates are still broadly consistent with the mid-Pliocene abrupt uplift of the eastern Tibetan Plateau and adjacent southwest China (*c*. 3.4 Ma) [102], and the intensification of Northern Hemisphere Glaciation (3.2–2.5 Ma) [103]. These geological and/or climatic changes had possibly acted as an isolating barrier between regional populations (i.e. western China vs central/eastern China; south Japan vs central Japan), and promoted the diversification of two lineages of *E. pleiosperma* (western vs central/eastern) and two phylogroups of *E. polyandra* (J1 vs J2). In fact, such Late Neogene tectonic/climate-induced vicariance has also been invoked to explain phylogeographic splits in other Tertiary-relic deciduous tree species from East Asia (e.g. *Cercidiphyllum* spp.; *Tetracentron sinense*; *Davidia involucrata*; *Sargentodoxa cuneata*) [3, 4, 12, 104]. However, our estimated coalescent times of the various cpDNA phylogroups within *E. pleiosperma* (nodes C1 and C2: 1.66 Ma; C3: 1.15 Ma; C4: 1.73 Ma) and *E. polyandra* (node J1: 1.50 Ma; J2: 2.31 Ma) are generally more recent (Fig. 4b; Table 2), implying that this haplotype diversity and structure was most likely shaped by historical processes during the (Late) Quaternary (see below).

### Relative demographic stability of *E. pleiosperma* in China

In contrast with the profound influence of Quaternary climatic fluctuations on the range dynamics of both *Cercidiphyllum japonicum* [3] and *Sargentodoxa cuneata* [104] in China, our results suggest a rather limited effect of such climate change on the demographic history and LGM distribution of *E. pleiosperma* in this region. This inference is based on three lines of evidence. First, *E. pleiosperma* exhibits strong phylogeographic structure in cpDNA (*N*_ST_ = 0.847 > *G*_ST_ = 0.835, *P* < 0.05), which largely reflects the presence of four montane phylogroups, one in the west (C1), and three (C2–4) in the Central-East (Figs. 1 and 4b). In fact, the only evident instances of inter-region seed flow pertain to the occurrence of both a western (H9) and C2 chlorotype (H13) in a single population (TS) from the SB region (Fig. 1); this is probably best explained by two independent migration events from the western/mid-lower Yangtze River regions, followed by the extinction of H9 and H13 in their respective source areas. Second, the BSP analysis for *E. pleiosperma* (Additional file 8: Figure S3a) indicates population growth up to the Late Quaternary (*c.* 0.25 Ma), followed by population stability until the present. Finally, our ENM for the LGM (Fig. 5b) suggests only slightly larger and more contiguous distributions of *E. pleiosperma*, particularly in areas around the Sichuan Basin and, to a lesser extent, along the northern flanks of the Yangtze River, when compared to the LIG or the present (Fig. 5a and c).

Together, these cpDNA data suggest that populations of *E. pleiosperma* largely persisted in their separate mountainous refugia over periods of Quaternary climate change up to the present [4, 12, 105]. Such population persistence might have been facilitated through periodic episodes of up-slope contraction during interglacials, and down-slope expansion during glacials, with populations tracking favourable humidity conditions as imposed by the East Asian monsoon in areas of high relief [106]. During glacials, genetic exchange between neighboring montane lineages might have occurred through lower-elevation populations, as has been suggested for other mid- and high-elevation plant and animal taxa elsewhere [107–110]. In fact, the majority of *E. pleiosperma* populations belonging to the three central-eastern phylogroups share the same nuclear (nSSR) gene pool (II), excepting three from the SB region (DF, HS, YN) that share gene pool I with most western populations (Fig. 3). This pattern of nuclear cohesion, which is also reflected in the ITS data (Fig. 2), is thus not entirely unexpected if we assume that inter-region gene exchange during glacials mainly occurred via pollen in low-elevation populations that were also probably the first to be extirpated during elevation shifts caused by postglacial climate warming [110, 111]. However, when compared with the situation in the Central-East, it also appears (at least from the nSSR data) that pollen less readily dispersed between the western and central-eastern phylogroups; this is most likely caused by topographical effects of the eastern QTP/Hengduan Mts. rather than the minor (and overall non-significant) differences in eco-climatic space found between these groups (Additional file 10: Figure S5; Fig. 7). Notably, this most evident genetic break within *E. pleiosperma* around the Sichuan Basin coincides with the boundary between the Sino-Himalayan and Sino-Japanese Forest subkingdoms [112], and is also apparent in several other plant taxa [10, 113, 114].

### Quaternary range shifts and cryptic glacial survival of *E. polyandra* in Japan

Our cpDNA results show that *E. polyandra* is comprised of two phylogroups with unique sets of chlorotypes and distinct geographic distributions, one in south Japan (Kyushu, Shikoku, south Honshu/Chubu) and the other in central Japan (north Honshu) (Fig. 1a, b). Given that *E. polyandra* is a moisture-dependent and drought-sensitive species, the deterioration of Tertiary warm, moist climates during the mid-to-late Pliocene (see above) likely triggered ecological changes [115], and hence drove the diversification of *E. polyandra*. Accordingly, this phylogeographic break in central Honshu, which previously has also been observed in several other biota [96, 116–118], must have been maintained in spite of an apparently profound influence of Late Quaternary climate change on the species demographic history and distribution. The latter is inferred from two lines of evidence. The first is the BSP analysis, which indicates that *E. polyandra* (and its phylogroups) experienced long-term demographic stability before *N*_e_ started to increase only relatively recently, *c*. 0.5 Ma (Additional file 8: Figure S3b–d). This population growth may have been triggered by climate change at the beginning of China’s ‘Penultimate Interglacial Period’ (*c*. 0.46–0.33 Ma) [119] and the intensification of the warm, wet summer monsoon in East Asia since the mid-late Pleistocene (*c*. 1.0–0.78 Ma) [120]. For moisture-dependent island trees, such as *E. polyandra* [99], this may have also led to an increase of suitable habitats throughout the warmer periods of the Late Quaternary. The second line of evidence comes from our ENM (Fig. 5), suggesting that, during the LGM, *E. polyandra* experienced a dramatic contraction of its (present-like) LIG range into a narrow coastal strip along the Pacific Ocean side of southeast Japan (Fig. 5). Notably, the single population surveyed from Kyushu (MI) harbours an phylogenetically basal chlorotype (H31) that also predominates in south Honshu, whereas its mutational derivative (H34) is private to Shikoku (Fig. 1a and b). It is feasible, therefore, that southeast Japan (most likely Kyushu) provided the source populations for postglacial (re-)colonization of these latter areas. Supportive evidence for this scenario comes from the fossil pollen record, demonstrating an increase of WTD forest in south Honshu/Chugoku during early postglacial times [121]. However, in contrast to the ENM results, there are two lines of genetic evidence for the glacial survival of *E. polyandra* in north Honshu: first, this region exclusively harbours private cpDNA haplotypes (Fig. 1a and b), and second, based on the nSSRs, there is no decreasing trend of genetic diversity (in terms *A*_R_, *PA*_R_) compared with areas further south (see Additional file 2: Table S1), as would be expected under a leading edge model of northward colonization [122]. Rather, these data suggest that extant populations in north Honshu are derived from a cryptic glacial refuge that occurred within local favourable microclimates [123], but which cannot be detected by ENM-based palaeodistribution reconstructions [85, 124]. Evidently, both nuclear markers (nSSR, ITS) failed to detect this refuge, and hence the south-central divide of *E. polyandra* in cpDNA (Figs. 1, 2 and 3). It appears unlikely that this discordance reflects incomplete nuclear lineage sorting, given the timeframe of the phylogroup divergence. Rather, a more tenable explanation is that the species’ range-wide nuclear cohesion results from periodic secondary contact and admixture via pollen during inter-/postglacials.

## Conclusions

The speciation and lineage diversification events of *Euptelea* reflect the primary influence of successive climate/tectonic-induced vicariance during the Late Miocene and Pliocene periods. By contrast, the haplotype diversity and structure at the within-group level of each *Euptelea* species are most likely shaped by the climatic cycles of the (Late) Quaternary. During this time, *E. pleiosperma* populations seem to have been mostly stationary within their multiple mountain refugia, whereby topographical constraints to seed/pollen dispersal fostered the divergence among ‘western’ and ‘central-eastern’ cpDNA phylogroups. Contemporaneously, *E. polyandra* populations in south Japan apparently underwent repeated range contractions/expansions, while those in central Japan may have persisted in a cryptic glacial refuge in north Honshu. Overall, our results (1) illustrate how Late Neogene climatic changes promoted speciation and lineage diversification in East Asia’s Tertiary relict flora; and (2) demonstrate for the first time a greater variation in such species’ responses to glacial cycles in Japan when compared to congeners in China. Overall, this study should contribute to a better understanding of the potential historical (e.g. climatic, geomorphological) processes that may have generally impacted the East Asia’s Arcto-Tertiary flora.

## Availability of supporting data

DNA sequences: see Genbank accessions for *E. pleiosperma and E. polyandra* in Additional file 3: Table S2 in supporting information). In addition, The sequence alignments and phylogenetic trees were deposited in TreeBASE (http://www.treebase.org/) under the submission number 18919 (URL: http://purl.org/phylo/treebase/phylows/study/TB2:S18919).

## Additional files

Additional file 1: Figure S1. (a) Map showing the location of places quoted in the text including 26 sampled populations of *E. pleiosperma* (red dots) and 10 sampled populations of *E. polyandra* (blue dots). More information about the sampled populations is available in Additional file 2: Table S1. (b) Fossil records of extinct and potentially extant (‘*Euptelea*-like’) species of Eupteleaceae in North America and East Asia [2]. (PDF 1037 kb) Additional file 2: Table S1. Geographic and genetic characteristics of 26 *E. pleiosperma* and 10 *E. polyandra* populations used in this study. (DOC 130 kb) Additional file 3: Table S2. GenBank accession numbers of newly obtained cpDNA and ITS sequences (prefix ‘KR’), and some downloaded sequences from GenBank used in the present study. (DOC 158 kb) Additional file 4: Table S3. Chloroplast DNA sequence polymorphisms detected in *Euptelea* at two IGS and one intron regions, identifying 35 chlorotypes (H1–35). (DOC 251 kb) Additional file 5: Table S4. ITS sequence polymorphisms detected in *Euptelea* at ITS1 and ITS2 (the 5.8S excluded) regions and identifying 10 ribotypes (R1–10). (DOC 63 kb) Additional file 6: Table S5. Mean estimates of genetic diversity and differentiation at eight nuclear microsatellite loci surveyed across 33 *Euptelea* populations (marked with an asterisk in Additional file 2: Table S1). (DOC 30 kb) Additional file 7: Figure S2.
structure results for identifying the optimal number of clusters (*K*) among 440 individuals (36 populations) of *Euptelea* based on nSSR data. The number of *K* was varied from 1 to 36 in 10 independent runs. The dot plot indicates the mean posterior probability [lnP(*D*)] for each value of *K* [64], and the superimposed line diagram represents the corresponding Δ*K* statistics calculated according to Evanno et al. [65]. (PDF 337 kb) Additional file 8: Figure S3. Bayesian Skyline Plots (BSPs) inferred from cpDNA (*psb*A–*trn*H, *rpo*B–*trn*C, *rpL16*, *pet*N*–trn*C, *mat*K, *rbc*L) sequences, and depicting changes of effective population size (*N*_e_; y–axis) through time [in million of years ago (Ma); x–axis] in: (a) *E. pleiosperma*; (b) *E. polyandra*; and (c) the J1 and (d) the J2 phylogroups of the latter species. The black solid line is the median estimate, and the gray dashed line marks the 95 % highest probability density (HPD) intervals. (PDF 475 kb) Additional file 9: Figure S4. Box-plots of 19 BIOCLIM variables and results of *t*-tests for 26 populations of *E. pleiosperma* in China and 10 populations of *E. polyandra* in Japan. Each box plot shows the maximum, 75th percentile, 25th percentile, and minimum value around the median value for each species. (PDF 443 kb) Additional file 10: Figure S5. Box-plots of 19 BIOCLIM variables and results of *t*-tests for two cpDNA lineages of *E. pleiosperma* in China (W: western populations; CE: central-eastern populations). Each box plot shows the maximum, 75th percentile, 25th percentile, and minimum value around the median value for each population group (‘lineage’). (PDF 392 kb)
