# Supplementary figures and images for: Evolution of East Asia’s Arcto-Tertiary relict Euptelea (Eupteleaceae) shaped by Late Neogene vicariance and Quaternary climate change
Source: BMC Evol Biol. 2016 Mar 22;16:66. doi: 10.1186/s12862-016-0636-x (PMC4802896; doi:10.1186/s12862-016-0636-x)

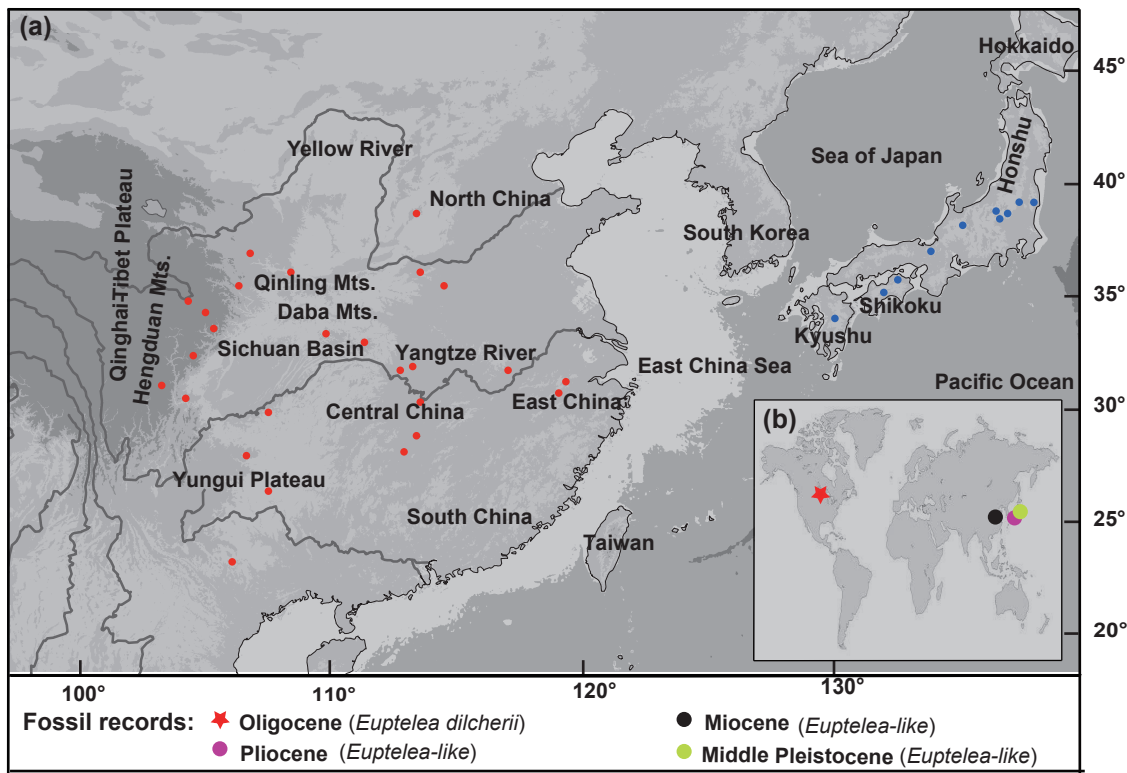

Supplement: Additional file 1: Figure S1. — (a) Map showing the location of places quoted in the text including 26 sampled populations of E. pleiosperma (red dots) and 10 sampled populations of E. polyandra (blue dots). More information about the sampled populations is available in Additional file 2: Table S1. (b) Fossil records of extinct and potentially extant (‘Euptelea-like’) species of Eupteleaceae in North America and East Asia [2]. (PDF 1037 kb) [file 12862_2016_636_MOESM1_ESM.pdf]

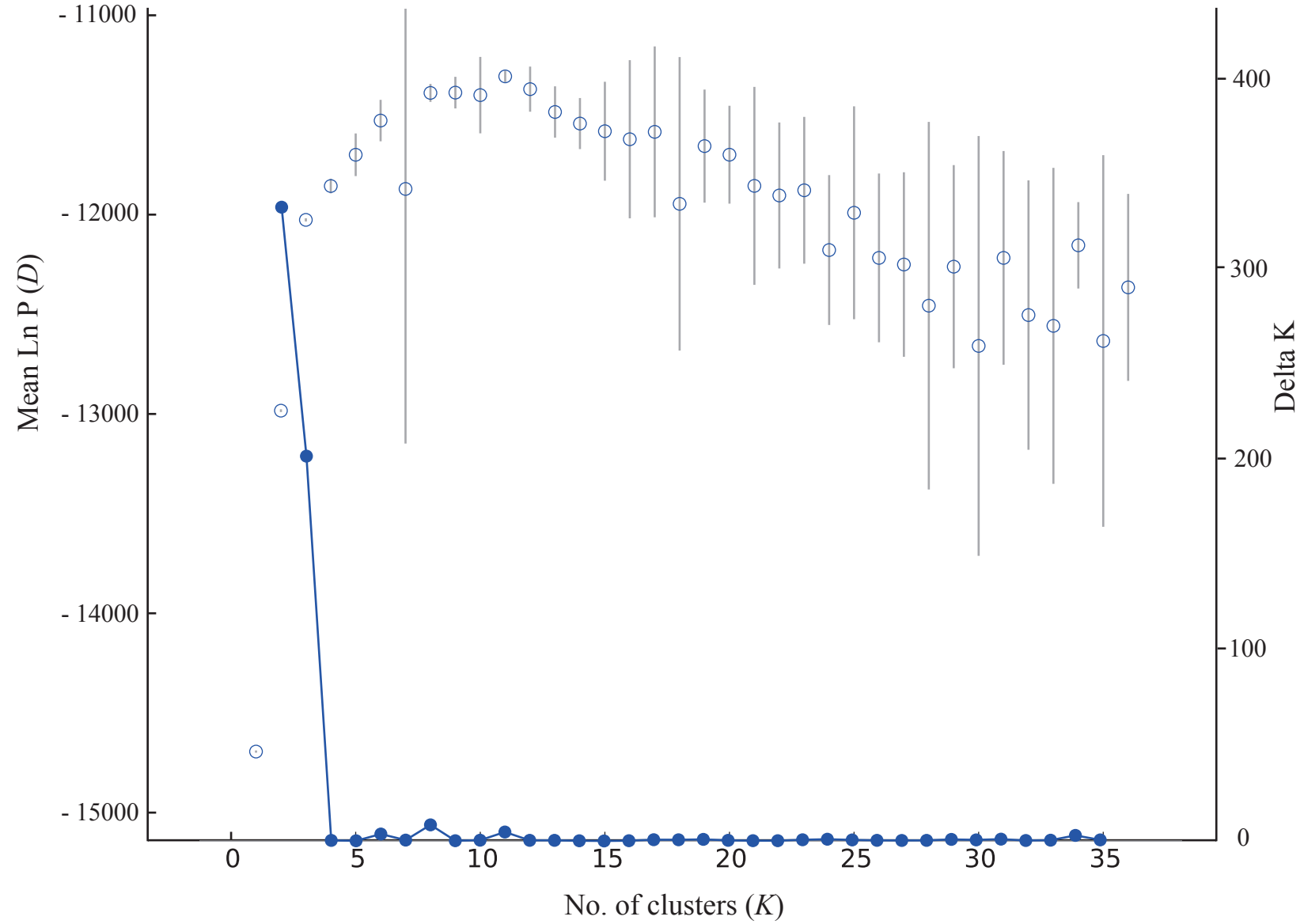

Supplement: Additional file 7: Figure S2. — structure results for identifying the optimal number of clusters (K) among 440 individuals (36 populations) of Euptelea based on nSSR data. The number of K was varied from 1 to 36 in 10 independent runs. The dot plot indicates the mean posterior probability [lnP(D)] for each value of K [64], and the superimposed line diagram represents the corresponding ΔK statistics calculated according to Evanno et al. [65]. (PDF 337 kb) [file 12862_2016_636_MOESM7_ESM.pdf]

(a)

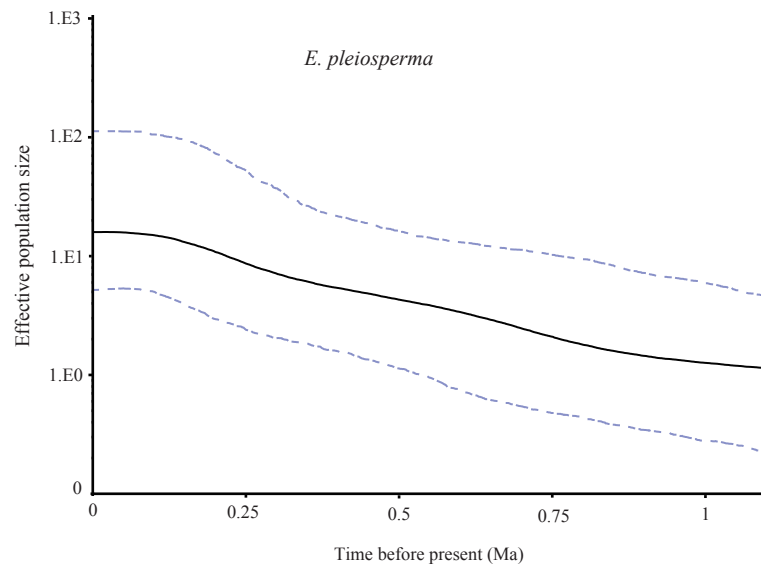

(b)

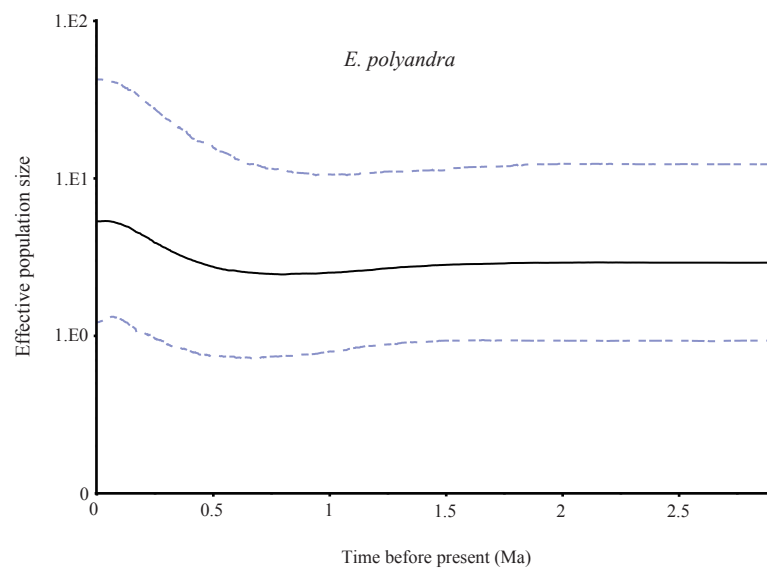

(c)

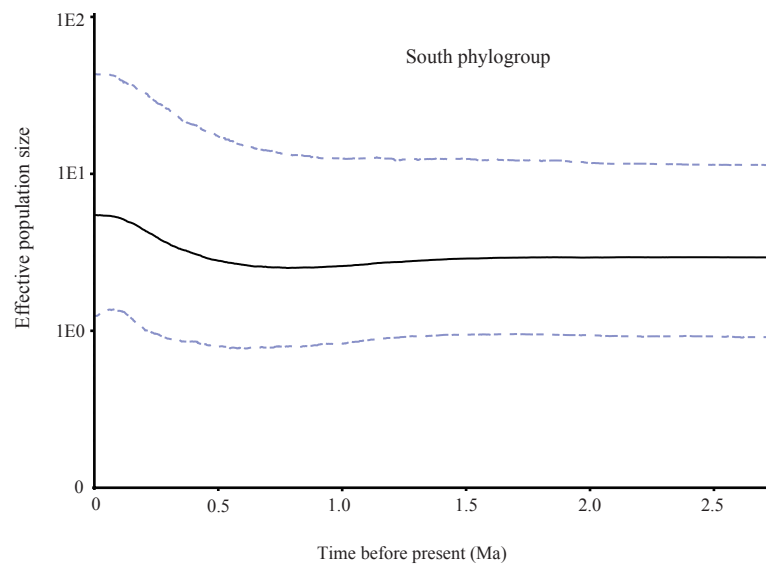

(d)

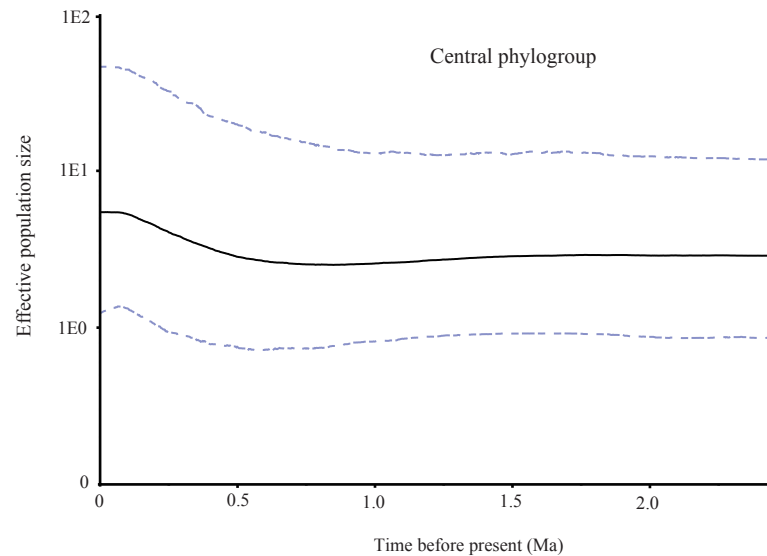

Supplement: Additional file 8: Figure S3. — Bayesian Skyline Plots (BSPs) inferred from cpDNA (psbA–trnH, rpoB–trnC, rpL16, petN–trnC, matK, rbcL) sequences, and depicting changes of effective population size (N e; y–axis) through time [in million of years ago (Ma); x–axis] in: (a) E. pleiosperma; (b) E. polyandra; and (c) the J1 and (d) the J2 phylogroups of the latter species. The black solid line is the median estimate, and the gray dashed line marks the 95 % highest probability density (HPD) intervals. (PDF 475 kb) [file 12862_2016_636_MOESM8_ESM.pdf]

BIO1

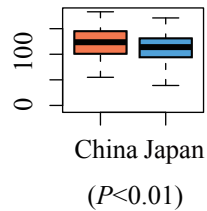

BIO2

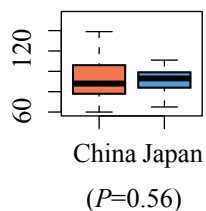

BIO3

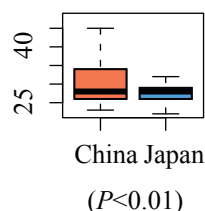

BIO4

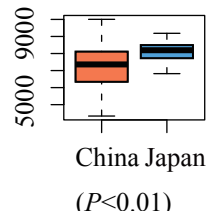

BIO5

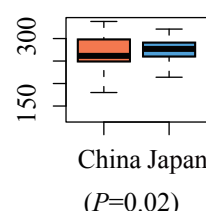

BIO6

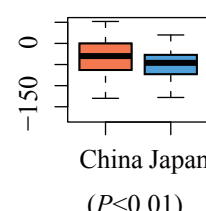

BIO7

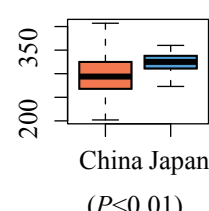

BIO8

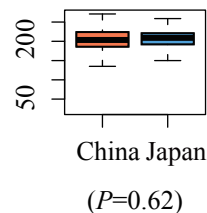

BIO9

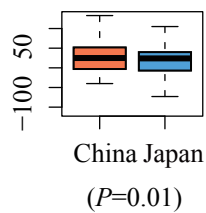

BIO10

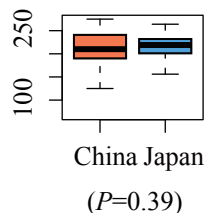

BIO11

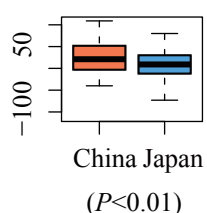

BIO12

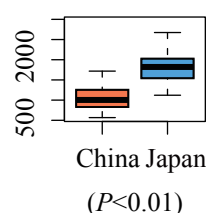

BIO13

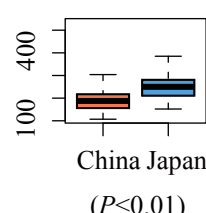

BIO14

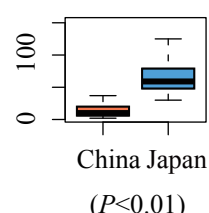

BIO15

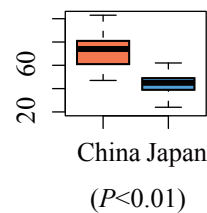

BIO16

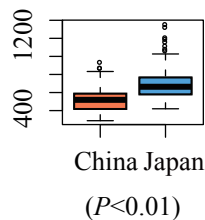

BIO17

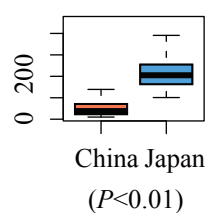

BIO18

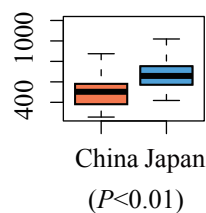

BIO19

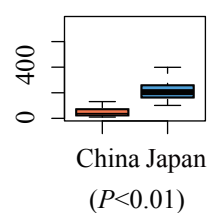

Supplement: Additional file 9: Figure S4. — Box-plots of 19 BIOCLIM variables and results of t-tests for 26 populations of E. pleiosperma in China and 10 populations of E. polyandra in Japan. Each box plot shows the maximum, 75th percentile, 25th percentile, and minimum value around the median value for each species. (PDF 443 kb) [file 12862_2016_636_MOESM9_ESM.pdf]

BIO1

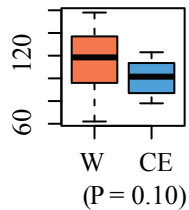

BIO2

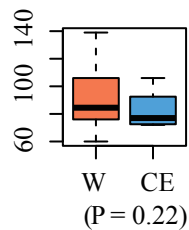

BIO3

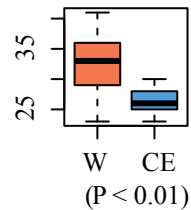

BIO4

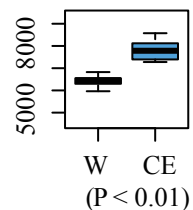

BIO5

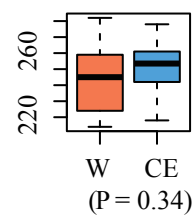

BIO6

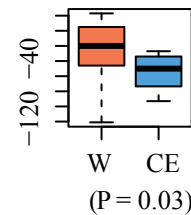

BIO7

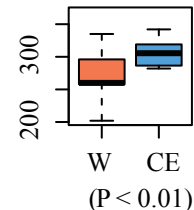

BIO8

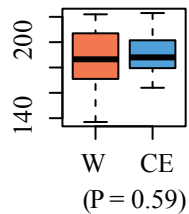

BIO9

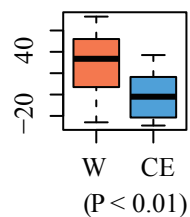

BIO10

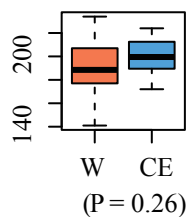

BIO11

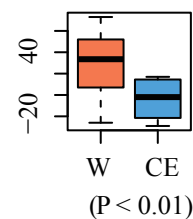

BIO12

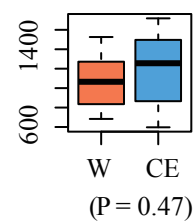

BIO13

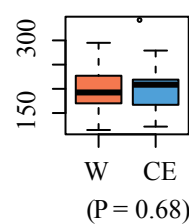

BIO14

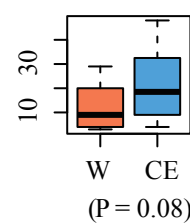

BIO15

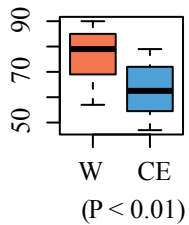

BIO16

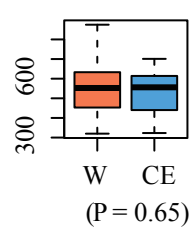

BIO17

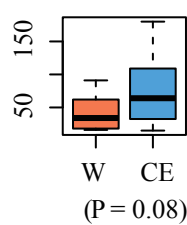

BIO18

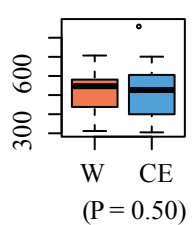

BIO19

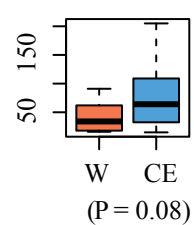

Supplement: Additional file 10: Figure S5. — Box-plots of 19 BIOCLIM variables and results of t-tests for two cpDNA lineages of E. pleiosperma in China (W: western populations; CE: central-eastern populations). Each box plot shows the maximum, 75th percentile, 25th percentile, and minimum value around the median value for each population group (‘lineage’). (PDF 392 kb) [file 12862_2016_636_MOESM10_ESM.pdf]
