# Supplementary material for: Evolution of East Asia’s Arcto-Tertiary relict Euptelea (Eupteleaceae) shaped by Late Neogene vicariance and Quaternary climate change
Source: BMC Evol Biol. 2016 Mar 22;16:66. doi: 10.1186/s12862-016-0636-x (PMC4802896; doi:10.1186/s12862-016-0636-x)
Supplement: Additional file 2: Table S1. — Geographic and genetic characteristics of 26 E. pleiosperma and 10 E. polyandra populations used in this study. (DOC 130 kb) [file 12862_2016_636_MOESM2_ESM.doc]

**Additional file 1: Table S1.** Geographic and genetic characteristics of 26 *E. pleiosperma* and 10 *E. polyandra* populations used in this study.

|  |  | |  |  |  |  | cpDNA |  |  | ITS |  |  | nSSR |  |
| --- | --- | --- | --- | --- | --- | --- | --- | --- | --- | --- | --- | --- | --- | --- |
| Species/ Population code | Locations | | Latitude (°N)/ Longitude (°E) | Altitude(m) | *n* (cpDNA/ITS/nSSR) | Haplotype distribution (cpDNA/ITS) | *h* | *π* × 10^-3^ |  | *h* | π × 10^-3^ |  | *PA_R_* | *A_R_* |
| ***E. pleiosperma* (China)** | |  | | | | | | | | | | | | |
| TQ^＊^ | Mt. Erlangshan, Sichuan | | 29.89/102.353 | 1694–1906 | 10/4/20 | H6,H8/R1–3    H19/R1–3 | 0.556 | 0.55 |  | 0.833 | 3.98 |  | 0 | 5 |
| EM^＊^ | Mt. Emei, Sichuan | | 29.588/103.333 | 963–1879 | 13/5/14 | H6, H7/R1, R3 | 0.282 | 0.14 |  | 0.4 | 1.59 |  | 0 | 4.86 |
| DY^＊^ | Dujiangyan, Sichuan | | 31.119/103.562 | 1500 | 10/5/22 | H3–5/R1–3 | 0.644 | 0.89 |  | 0.8 | 3.98 |  | 0.16 | 5.21 |
| SJ^＊^ | Pingwu, Sichuan | | 32.18/104.325 | 1009–1422 | 9/5/26 | H1/R1–2 | 0 | 0 |  | 0.4 | 1.59 |  | 0.11 | 5.36 |
| BM^＊^ | Pingwu, Sichuan | | 32.681/104.431 | 1514 | 4/4/8 | H1–2/R1 | 0.5 | 0.25 |  | 0 | 0 |  | 0.03 | 6.13 |
| JZ^＊^ | Jiuzhaigou, Sichuan | | 33.192/103.906 | 2187–2337 | 4/4/11 | H1/R1 | 0 | 0 |  | 0 | 0 |  | 0.09 | 5.06 |
| TT | Tiantangzhai, Anhui | | 31.132/115.776 | 982 | 1/4/5 | H23/R1, R5 | － | － |  | 0.5 | 3.98 |  | － | － |
| BT^＊^ | Baotianman, Henan | | 33.49/111.929 | 1231 | 7/5/11 | H23, H25/R1 | 0.286 | 0.42 |  | 0 | 0 |  | 0.15 | 5.95 |
| LC^＊^ | Luanchuan, Henan | | 33.891/111.616 | 1210 | 9/5/18 | H23/R1 | 0 | 0 |  | 0 | 0 |  | 0.2 | 5.56 |
| DJ^＊^ | Shennongjia, Hubei | | 31.447/110.149 | 1772–1839 | 10/4/13 | H23/R1–2 | 0 | 0 |  | 0.5 | 1.99 |  | 0.05 | 5.85 |
| LJ^＊^ | Shennongjia, Hubei | | 31.554/110.35 | 1647–1713 | 7/1/12 | H23/R1 | 0 | 0 |  | － | － |  | 0.07 | 5.78 |
| YC^＊^ | Dalaoling, Hubei | | 31.08/110.92 | 1532–1692 | 6/2/11 | H19–20/R1 | 0.533 | 0.79 |  | 0 | 0 |  | 0.05 | 5.84 |
| DB^＊^ | Mt. Daba, Chongqing | | 32.141/108.58 | 1145–1188 | 9/5/15 | H23–24/R1 | 0.556 | 0.27 |  | 0 | 0 |  | 0.01 | 6.35 |
| JF^＊^ | Mt. Jinfo, Chongqing | | 29.037/107.186 | 1733–1928 | 4/4/9 | H16/R1 | 0 | 0 |  | 0 | 0 |  | 0.11 | 6.97 |
| HS^＊^ | Huishui, Guizhou | | 26.066/106.968 | 922–1070 | 6/3/11 | H10–12/R1–2 | 0.6 | 0.43 |  | 0.667 | 2.66 |  | 0.14 | 4.47 |
| DF^＊^ | Dafang, Guizhou | | 27.407/105.903 | 1518–1657 | 10/4/15 | H10, H16–17/R1–2 | 0.511 | 0.63 |  | 0.5 | 1.99 |  | 0.2 | 5.16 |
| HP^＊^ | Mt. Huping, Hunan | | 30.039/110.531 | 1304–1845 | 9/6/16 | H21–22/R1 | 0.389 | 0.19 |  | 0 | 0 |  | 0.08 | 5.79 |
| TP^＊^ | Mt. Tianping, Hunan | | 29.758/110.062 | 1231 | 7/4/11 | H18/R1 | 0 | 0 |  | 0 | 0 |  | 0 | 3.11 |
| ZP^＊^ | Zhenping, Shaanxi | | 32.006/109.293 | 1923 | 11/5/20 | H24/R1 | 0 | 0 |  | 0 | 0 |  | 0.05 | 5.69 |
| FP^＊^ | Foping, Shaanxi | | 33.667/107.969 | 1300 | 8/5/11 | H23/R1 | 0 | 0 |  | 0 | 0 |  | 0.2 | 5.41 |
| QL^＊^ | Qingliangfeng, Zhejiang | | 30.134/118.865 | 836–878 | 8/4/10 | H21/R1, R4 | 0 | 0 |  | 0.667 | 2.66 |  | 0 | 3.83 |
| TM^＊^ | Mt. Tianmu, Zhejiang | | 30.36/119.428 | 1016 | 8/5/11 | H21/R1 | 0 | 0 |  | 0 | 0 |  | 0.13 | 4.33 |
| TS^＊^ | Tianshui, Gansu | | 34.303/106.107 | 1582–1821 | 10/5/12 | H9,H13/R1–2 | 0.2 | 0.49 |  | 0.4 | 1.59 |  | 0 | 5.89 |
| KX^＊^ | Kangxian, Gansu | | 33.369/105.49 | 1474–1562 | 9/5/16 | H10/R1 | 0 | 0 |  | 0 | 0 |  | 0.07 | 5.51 |
| YN^＊^ | Malipo, Yunnan | | 23.156/104.829 | 1743 | 7/5/14 | H14–15/R2 | 0.286 | 0.42 |  | 0 | 0 |  | 0.01 | 3.77 |
| SX^＊^ | Xiaxian, Shanxi | | 34.974/111.428 | 840–914 | 8/5/8 | H23/R1–3 | 0 | 0 |  | 0.7 | 3.19 |  | 0 | 5 |
| **Species mean** |  | |  |  |  |  | **0.214** | **0.219** |  | **0.255** | **1.168** |  | **0.08** | **5.275** |
| **Species total** |  | |  |  |  |  | **0.893** | **1.73** |  | **0.375** | **1.73** |  | **－** | **－** |
| ***E. polyandra* (Japan)** | |  | | | | | | | | | | | | |
| FU^＊^ | Iwaki, Fukushima | | 36.91/140.72 |  | 10/4/9 | H26–27/R6, R9–10 | 0.2 | 0.1 |  | 0.833 | 3.98 |  | 0 | 3.68 |
| SA^＊^ | Chichibu, Saitama | | 35.917/138.831 |  | 8/5/9 | H26–27/R6, R10 | 0.25 | 0.12 |  | 0.6 | 4.78 |  | 0.07 | 4.77 |
| RY^＊^ | Mima, Tokushima | | 34.11/134.042 |  | 10/5/14 | H34–35/R6, R10 | 0..356 | 0.18 |  | 0.4 | 3.19 |  | 0 | 3.54 |
| TA | Takaoka, Kochi | | 33.549/133.349 |  | 3/4/3 | H34/R6, R8, R10 | 0 | 0 |  | 0.833 | 4.65 |  | － | － |
| KK | Kibune, Kyoto | | 35.954/137.328 |  | 3/3/4 | H31/R6, R9 | 0 | 0 |  | 0.667 | 2.66 |  | － | － |
| KG^＊^ | Kosaka, Gifu | | 35.136/135.765 |  | 6/5/9 | H30–31/R10 | 0.533 | 0.26 |  | 0 | 0 |  | 0.11 | 3.88 |
| TK^＊^ | Takayama, Gifu | | 36.179/137.383 |  | 11/6/11 | H31/R9–10 | 0 | 0 |  | 0.533 | 2.12 |  | 0.09 | 3.25 |
| MA^＊^ | Matsumoto, Nagano | | 36.199/137.767 |  | 10/5/10 | H31/R6, R8–10 | 0 | 0 |  | 0.9 | 4.78 |  | 0 | 3.14 |
| IW^＊^ | Iwaki, Fukushima | | 36.927/140.664 |  | 8/5/8 | H26, H28–29/R7, R9–10 | 0.607 | 0.34 |  | 0.7 | 3.19 |  | 0.13 | 3.63 |
| MI^＊^ | Takachiho-cho, Miyazaki | | 32.797/131.270 |  | 13/6/13 | H31, H32–33/R6 | 0.41 | 0.22 |  | 0 | 0 |  | 0 | 3.21 |
| **Species mean** |  | |  |  |  |  | **0.2** | **0.122** |  | **0.547** | **2.94** |  | **0.05** | **3.638** |
| **Species total** |  | |  |  |  |  | **0.707** | **0.49** |  | **0.674** | **3.93** |  | **－** | **－** |

Only populations marked with an asterisk were used for analyses of genetic diversity and differentiation for nSSR (see text for further explanation). *n*, sample sizes for cpDNA/ITS/nSSR analyses; *h*, haplotype diversity; *π*, nucleotide diversity; *PA_R_*, private allelic richness; *A_R_*, allelic richness.
