# Supplementary material for: Evolution of East Asia’s Arcto-Tertiary relict Euptelea (Eupteleaceae) shaped by Late Neogene vicariance and Quaternary climate change
Source: BMC Evol Biol. 2016 Mar 22;16:66. doi: 10.1186/s12862-016-0636-x (PMC4802896; doi:10.1186/s12862-016-0636-x)
Supplement: Additional file 3: Table S2. — GenBank accession numbers of newly obtained cpDNA and ITS sequences (prefix ‘KR’), and some downloaded sequences from GenBank used in the present study. (DOC 158 kb) [file 12862_2016_636_MOESM3_ESM.doc]

**Additional file 2: Table S2.** GenBank accession numbers of newly obtained cpDNA and ITS sequences (prefix ‘KR’ ), and some downloaded sequences from GenBank used in the present study.

| GenBank accession numbers | | | | | | | | | | |
| --- | --- | --- | --- | --- | --- | --- | --- | --- | --- | --- |
| Taxon | Chlorotype | *psb*A-*trn*H | *rpL 16* | *rpo*B*-trn*C | *pet*N-*trn*C | *mat*K | *rbc*L |  | Ribotype | ITS |
| *E. pleiosperma* | H1 | KR064689 | KR064654 | KR064619 | KR064725 | KR232452 | KR232415 |  | R1 | KR011726 |
|  | H2 | KR064690 | KR064655 | KR064620 | KR064726 | KR232453 | KR232416 |  | R2 | KR011727 |
|  | H3 | KR064697 | KR064662 | KR064627 | KR064733 | KR232460 | KR232423 |  | R3 | KR011728 |
|  | H4 | KR064698 | KR064663 | KR064628 | KR064734 | KR232461 | KR232424 |  | R4 | KR011729 |
|  | H5 | KR064699 | KR064664 | KR064629 | KR064735 | KR232462 | KR232425 |  | R5 | KR011730 |
|  | H6 | KR064700 | KR064665 | KR064630 | KR064736 | KR232463 | KR232426 |  | R6 | KR011731 |
|  | H7 | KR064701 | KR064666 | KR064631 | KR064737 | KR232464 | KR232427 |  | R7 | KR011732 |
|  | H8 | KR064707 | KR064672 | KR064637 | KR064743 | KR232480 | KR232443 |  | R8 | KR011733 |
|  | H9 | KR064709 | KR064674 | KR064639 | KR064745 | KR232482 | KR232445 |  | R9 | KR011734 |
|  | H10 | KR064694 | KR064659 | KR064624 | KR064730 | KR232457 | KR232420 |  | R10 | KR011735 |
|  | H11 | KR064704 | KR064669 | KR064634 | KR064740 | KR232469 | KR232432 |  |  |  |
|  | H12 | KR064705 | KR064670 | KR064635 | KR064741 | KR232470 | KR232433 |  |  |  |
|  | H13 | KR064708 | KR064673 | KR064638 | KR064744 | KR232481 | KR232444 |  |  |  |
|  | H14 | KR064712 | KR064677 | KR064642 | KR064748 | KR232485 | KR232448 |  |  |  |
|  | H15 | KR064713 | KR064678 | KR064643 | KR064749 | KR232486 | KR232449 |  |  |  |
|  | H16 | KR064702 | KR064667 | KR064632 | KR064738 | KR232458 | KR232421 |  |  |  |
|  | H17 | KR064696 | KR064661 | KR064626 | KR064732 | KR232459 | KR232422 |  |  |  |
|  | H18 | KR064706 | KR064671 | KR064636 | KR064742 | KR232479 | KR232442 |  |  |  |
|  | H19 | KR064710 | KR064675 | KR064640 | KR064746 | KR232483 | KR232446 |  |  |  |
|  | H20 | KR064711 | KR064676 | KR064641 | KR064747 | KR232484 | KR232447 |  |  |  |
|  | H21 | KR064703 | KR064668 | KR064633 | KR064739 | KR232468 | KR232431 |  |  |  |
|  | H22 | KR064695 | KR064660 | KR064625 | KR064731 | KR232467 | KR232430 |  |  |  |
|  | H23 | KR064691 | KR064656 | KR064621 | KR064727 | KR232454 | KR232417 |  |  |  |
|  | H24 | KR064693 | KR064658 | KR064623 | KR064729 | KR232456 | KR232419 |  |  |  |
|  | H25 | KR064692 | KR064657 | KR064622 | KR064728 | KR232455 | KR232418 |  |  |  |
| *E. polyandra* | H26 | KR064714 | KR064679 | KR064644 | KR064750 | KR232465 | KR232428 |  |  |  |
|  | H27 | KR064715 | KR064680 | KR064645 | KR064751 | KR232466 | KR232429 |  |  |  |
|  | H28 | KR064716 | KR064681 | KR064646 | KR064752 | KR232471 | KR232434 |  |  |  |
|  | H29 | KR064717 | KR064682 | KR064647 | KR064753 | KR232472 | KR232435 |  |  |  |
|  | H30 | KR064718 | KR064683 | KR064648 | KR064754 | KR232473 | KR232436 |  |  |  |
|  | H31 | KR064719 | KR064684 | KR064649 | KR064755 | KR232474 | KR232437 |  |  |  |
|  | H32 | KR064720 | KR064685 | KR064650 | KR064756 | KR232475 | KR232438 |  |  |  |
|  | H33 | KR064721 | KR064686 | KR064651 | KR064757 | KR232476 | KR232439 |  |  |  |
|  | H34 | KR064722 | KR064687 | KR064652 | KR064758 | KR232477 | KR232440 |  |  |  |
|  | H35 | KR064723 | KR064688 | KR064653 | KR064759 | KR232478 | KR232441 |  |  |  |
| *Platanus occidentalis* |  | KR064761 | KR064760 | KR064724 | KR064618 | KR232451 | KR232450 |  |  |  |
| GenBank downloaded species |  | *rbc*L | *mat*K | 26S |  |  |  |  |  |  |
| *Aconitum racemulosum* Franch. |  | AY954488 | FJ626484 | AY954473 |  |  |  |  |  |  |
| *Aquilegia ecalcarata* Maxim. |  | AY954495 | EF437127 | AY954481 |  |  |  |  |  |  |
| *Asteropyrum cavaleriei* (L e´ vl. et Vant.) Drumm. & Hutch. |  | AF079453 | FJ626490 | AY954466 |  |  |  |  |  |  |
| *Caltha palustris* L. |  | L02431 | AB069845 | U52632 |  |  |  |  |  |  |
| *Clematis ganpiniana* (L e´ vl. & Vant.) Tamura |  | AY954491 | FJ626495 | AY954476 |  |  |  |  |  |  |
| *Coptis chinensis* Franch. |  | AY954497 | DQ478614 | AY954482 |  |  |  |  |  |  |
| *Glaucidium palmatum* Sieb. & Zucc. |  | AF093723 | AB069850 | AF389267 |  |  |  |  |  |  |
| *Helleborus thibetanus* Franch. |  | AY954485 | FJ626500 | AY954470 |  |  |  |  |  |  |
| *Hydrastis canadensis* L. |  | AF093725 | AB069849 | AF389268 |  |  |  |  |  |  |
| *Myosurus minimus* L. |  | DQ099441 | FJ626502 | FJ626447 |  |  |  |  |  |  |
| *Ranunculus cantoniensis* DC. |  | AY954489 | FJ626506 | AY954474 |  |  |  |  |  |  |
| *Thalictrum javanicum* Bl. |  | AY954496 | DQ478615 | AY954480 |  |  |  |  |  |  |
| *Xanthorhiza simplicissima* Marshall |  | L12669 | AB069848 | AF389270 |  |  |  |  |  |  |
| *Berberis thunbergii* DC. |  | AF139878 | AB069827 | FJ626454 |  |  |  |  |  |  |
| *Caulophyllum robustum* Maxim. |  | AF190441 | AB069832 | FJ626455 |  |  |  |  |  |  |
| *Caulophyllum thalictroides* (L.) Michx. |  | AF190442 | AB069831 | AF389240 |  |  |  |  |  |  |
| *Diphylleia cymosa* Michx. |  | L75866 | DQ478620 | FJ626456 |  |  |  |  |  |  |
| *Dysosma versipellis* (Hance) M. Cheng ex Ying |  | EF173669 | DQ478619 | FJ626458 |  |  |  |  |  |  |
| *Epimedium koreanum* Nakai |  | L75869 | AB069837 | FJ626459 |  |  |  |  |  |  |
| *Jeffersonia diphylla* (L.) Pers. |  | L75867 | AB069836 | U52604 |  |  |  |  |  |  |
| *Mahonia bealei* (Fort.) Carr. |  | L12657 | DQ478617 | FJ626461 |  |  |  |  |  |  |
| *Nandina domestica* Thunb. |  | L75843 | AB069830 | AF389241 |  |  |  |  |  |  |
| *Podophyllum peltatum* L. |  | AF093716 | AB069843 | DQ008614 |  |  |  |  |  |  |
| *Ranzania japonica* Ito |  | L75853 | AB069829 |  |  |  |  |  |  |  |
| *Sinopodophyllum hexandrum* (Royle) Ying |  | AF079455 | DQ478616 | FJ626463 |  |  |  |  |  |  |
| *Vancouveria hexandra* C. Morren & Decne. |  | EF173674 | AB069839 | U52602 |  |  |  |  |  |  |
| *Aspidocarya uvifera* Hook. f. & Thoms. |  | FJ626593 | EF143853 | FJ626465 |  |  |  |  |  |  |
| *Cyclea hypoglauca* (Schauer) Diels |  | FJ626594 | EF143862 | FJ626467 |  |  |  |  |  |  |
| *Pericampylus glaucus* (Lam.) Merr. |  | FJ626598 | EF143869 | FJ626471 |  |  |  |  |  |  |
| *Tinomiscium petiolare* Hook. f . & Thoms. |  | EF173675 | DQ478612 | FJ626474 |  |  |  |  |  |  |
| *Tinospora sinensis* (Lour.) Merr. |  | FJ626602 | EF143855 | FJ626475 |  |  |  |  |  |  |
| ***Euptelea pleiospermum* Hook. f. & Thoms.** |  | **AY048174** | **FJ626527** | **FJ626482** |  |  |  |  |  |  |
| ***Euptelea polyandra* Sieb. & Zucc.** |  | **L12645** | **FJ626528** | **AF389249** |  |  |  |  |  |  |
| *Meliosma veitchiorum* Hemsl. View. |  | AF206793 | FJ626530 | AF389271 |  |  |  |  |  |  |
| *Nelumbo lutea* (Willd.) Pers. |  | DQ182337 | AF543740 | AF389259 |  |  |  |  |  |  |
| *Nelumbo nucifera* Gaertn. |  | FJ626615 | FJ626531 | FJ626483 |  |  |  |  |  |  |
| *Platanus occidentalis* L. |  | L01943 | AF543747 | AF274662 |  |  |  |  |  |  |
| *Sabia swinhoei* Hemsl. |  | FJ626616 | FJ626532 | AF389272 |  |  |  |  |  |  |
