# Supplementary material for: Evolution of East Asia’s Arcto-Tertiary relict Euptelea (Eupteleaceae) shaped by Late Neogene vicariance and Quaternary climate change
Source: BMC Evol Biol. 2016 Mar 22;16:66. doi: 10.1186/s12862-016-0636-x (PMC4802896; doi:10.1186/s12862-016-0636-x)
Supplement: Additional file 4: Table S3. — Chloroplast DNA sequence polymorphisms detected in Euptelea at two IGS and one intron regions, identifying 35 chlorotypes (H1–35). (DOC 251 kb) [file 12862_2016_636_MOESM4_ESM.doc]

**Additional file 3: Table S3.** Chloroplast DNA sequence polymorphisms detected in *Euptelea* at two IGS and one intron regions, identifying 35 chlorotypes (H1–35).

| Chlorotype | Nucleotide position | | | | | | | | | | | | | | | | | | | | | | | | | | | | | | | | |
| --- | --- | --- | --- | --- | --- | --- | --- | --- | --- | --- | --- | --- | --- | --- | --- | --- | --- | --- | --- | --- | --- | --- | --- | --- | --- | --- | --- | --- | --- | --- | --- | --- | --- |
|  | *psb*A*-trn*H | | | | | |  | *rpL16* | | | | | |  | *rpo*B*-trn*C | | | | | | | | | | | | | | | | | | |
|  |  |  |  |  |  |  |  |  |  |  |  |  | 1 |  | 1 | 1 | 1 | 1 | 1 | 1 | 1 | 1 | 1 | 1 | 1 | 1 | 1 | 1 | 1 | 2 | 2 | 2 | 2 |
|  |  | 1 | 1 | 1 | 1 | 1 |  | 3 | 4 | 5 | 6 | 7 | 0 |  | 0 | 1 | 1 | 1 | 2 | 2 | 2 | 2 | 4 | 5 | 5 | 7 | 8 | 9 | 9 | 0 | 0 | 0 | 0 |
|  | 5 | 1 | 1 | 2 | 2 | 3 |  | 1 | 4 | 2 | 6 | 1 | 1 |  | 6 | 3 | 3 | 3 | 1 | 3 | 8 | 9 | 0 | 4 | 7 | 6 | 0 | 5 | 8 | 1 | 1 | 1 | 1 |
|  | 0 | 0 | 8 | 4 | 7 | 7 |  | 3 | 8 | 5 | 2 | 8 | 3 |  | 9 | 1 | 3 | 4 | 4 | 1 | 6 | 8 | 0 | 8 | 5 | 5 | 7 | 4 | 1 | 3 | 7 | 8 | 9 |
| H1 | 0^I^ | C | A | A | G | C |  | 1^b^ | C | G | A | C | A |  | T | T | T | T | G | G | A | T | A | C | A | A | C | C | T | T | A | C | T |
| H2 | 1^I^ | . | . | . | . | . |  | . | . | G | . | . | . |  | . | . | . | . | . | . | . | . | . | . | . | . | . | . | . | . | . | . | . |
| H3 | . | A | . | C | . | . |  | G | . | G | . | . | . |  | . | . | . | . | . | . | . | C | . | . | . | . | . | . | . | . | . | . | . |
| H3 | . | . | . | . | . | . |  | G | . | G | . | . | . |  | . | . | . | . | . | . | . | . | . | A | . | . | . | . | . | . | . | . | . |
| H4 | . | . | . | . | . | . |  | G | . | G | . | . | . |  | . | . | . | . | . | . | . | . | . | A | . | . | T | . | . | . | . | . | . |
| H5 | . | . | . | . | . | . |  | G | . | G | . | . | . |  | . | . | . | . | . | . | . | . | C | . | . | . | . | . | . | A | . | . | . |
| H6 | 1^I^ | . | . | . | A | . |  | G | . | G | . | . | . |  | . | . | . | . | T | . | . | . | . | . | . | . | . | . | . | . | . | . | . |
| H7 | 1^I^ | . | . | . | A | . |  | G | . | A | . | . | . |  | . | . | . | . | T | . | . | . | . | . | . | . | . | . | . | . | . | . | . |
| H8 | . | . | . | . | . | . |  | G | . | G | . | . | . |  | . | . | . | . | T | . | . | . | . | . | . | . | . | . | . | . | . | . | . |
| H9 | . | . | . | . | . | . |  | G | . | G | . | . | . |  | . | . | . | . | . | . | . | . | . | . | . | C | . | . | . | . | . | . | . |
| H10 | . | . | . | C | . | . |  | G | . | G | . | . | . |  | . | . | . | . | . | . | . | C | . | . | . | . | . | . | . | . | . | . | . |
| H11 | 1^I^ | . | . | C | . | . |  | G | . | G | . | . | . |  | . | . | . | . | . | . | . | C | . | . | . | . | . | . | . | . | . | . | . |
| H12 | 1^I^ | . | . | C | . | . |  | G | . | G | . | . | . |  | . | . | . | . | . | . | . | C | . | . | . | . | T | . | . | . | . | . | . |
| H13 | 1^I^ | . | . | C | . | 1^a^ |  | G | . | G | . | . | . |  | . | . | . | . | . | . | . | C | . | . | . | . | . | . | . | . | . | . | . |
| H14 | . | . | . | C | . | . |  | G | . | G | . | 1^c^ | . |  | . | . | . | . | . | . | . | C | . | . | . | . | . | . | . | . | . | . | . |
| H15 | . | . | . | C | . | . |  | G | . | G | . | 1^c^ | . |  | . | . | . | . | . | . | . | C | . | . | . | . | . | . | . | . | C | A | C |
| H16 | . | . | C | C | . | . |  | G | . | G | G | 1^c^ | . |  | . | . | . | . | . | . | . | C | . | . | . | . | . | . | . | . | . | . | . |
| H17 | . | . | C | C | . | . |  | G | . | G | G | 1^c^ | . |  | . | . | . | . | . | . | . | C | . | . | . | . | T | . | . | . | . | . | . |
| H18 | . | . | . | C | . | . |  | G | A | G | . | . | . |  | . | . | . | . | . | . | . | C | . | . | . | . | . | . | . | . | . | . | . |
| H19 | . | . | . | C | . | . |  | G | A | G | . | . | . |  | . | . | . | . | . | . | T | C | . | . | . | . | . | . | . | . | . | . | . |
| H20 | 1^I^ | . | . | C | . | . |  | G | A | G | . | . | . |  | . | . | . | . | . | 1^d^ | . | C | . | . | . | . | . | . | . | . | . | . | . |
| H21 | . | . | . | C | . | . |  | G | A | G | . | . | . |  | . | . | . | . | . | . | . | C | . | . | . | . | . | T | . | . | . | . | . |
| H22 | . | . | . | C | . | . |  | G | A | G | . | . | . |  | . | . | . | . | . | . | . | C | C | . | . | . | . | T | . | . | . | . | . |
| H24 | . | A | . | C | . | . |  | G | . | G | . | . | . |  | . | . | . | . | . | . | . | C | . | . | . | . | . | . | . | . | . | . | G |
| H25 | . | A | . | C | . | . |  | G | . | G | . | . | . |  | C | . | . | . | . | . | . | C | . | . | G | . | T | . | . | . | . | . | . |
| H26 | . | . | . | C | . | T |  | A | . | G | . | T | G |  | C | . | . | . | . | A | . | C | . | . | G | . | . | . | 1^e^ | . | . | . | . |
| H27 | . | . | . | C | . | T |  | A | . | G | . | T | G |  | C | . | . | . | . | A | . | C | . | . | G | . | T | . | 1^e^ | . | . | . | . |
| H28 | . | . | . | C | . | T |  | A | . | G | . | T | G |  | C | . | C | . | . | A | . | C | . | . | G | . | . | . | 1^e^ | . | . | . | . |
| H29 | . | . | . | C | . | T |  | A | . | G | . | T | G |  | C | C | . | . | . | A | . | C | . | . | G | . | . | . | 1^e^ | . | . | . | . |
| H30 | . | . | . | C | . | T |  | A | . | G | . | T | G |  | C | . | . | . | . | A | . | C | . | . | G | . | T | . | C | . | . | . | . |
| H31 | . | . | . | C | . | T |  | A | . | G | . | T | G |  | C | . | . | . | . | A | . | C | . | . | G | . | . | . | C | . | . | . | . |
| H32 | . | . | . | C | . | T |  | A | . | G | . | T | G |  | C | . | . | C | . | A | . | C | . | . | G | . | . | . | C | . | . | . | . |
| H33 | . | . | . | C | . | T |  | A | . | G | . | T | G |  | C | . | C | . | . | A | . | C | . | . | G | . | . | . | C | . | . | . | . |
| H34 | . | . | . | C | . | T |  | A | . | G | . | T | G |  | C | . | . | . | . | A | . | C | C | . | G | . | . | . | C | . | . | . | . |
| H35 | . | . | . | C | . | T |  | A | . | G | . | T | G |  | C | . | . | . | . | A | . | C | C | . | G | . | T | . | C | . | . | . | . |

Numbers 1/0 in sequences denote presence/absence of length polymorphism. Note that poly-A or poly-T stretches were excluded from analysis.

^a^ATGGTAGA, ^b^TATAAT, ^c^AATAA, ^d^TAGAAT, ^e^ATGGTTCCAATTTGCCCA,^I^AAAGAACAAGATAGTTCTCCCAC (inversion).
