# Supplementary material for: Evolution of East Asia’s Arcto-Tertiary relict Euptelea (Eupteleaceae) shaped by Late Neogene vicariance and Quaternary climate change
Source: BMC Evol Biol. 2016 Mar 22;16:66. doi: 10.1186/s12862-016-0636-x (PMC4802896; doi:10.1186/s12862-016-0636-x)
Supplement: Additional file 5: Table S4. — ITS sequence polymorphisms detected in Euptelea at ITS1 and ITS2 (the 5.8S excluded) regions and identifying 10 ribotypes (R1–10). (DOC 63 kb) [file 12862_2016_636_MOESM5_ESM.doc]

**Additional file 4: Table S4.** ITS sequence polymorphisms detected in *Euptelea* at ITS1 and ITS2 (the 5.8S excluded) regions and identifying 10 ribotypes (R1–10).

| Ribotype | Nucleotide position | | | | | | | | | | | | | | | | | | |
| --- | --- | --- | --- | --- | --- | --- | --- | --- | --- | --- | --- | --- | --- | --- | --- | --- | --- | --- | --- |
|  | ITS1 | | | | | | | | |  | ITS2 | | | | | | | | |
|  |  |  | 1 | 1 | 1 | 2 | 2 | 2 | 2 |  | 4 | 4 | 4 | 4 | 5 | 5 | 5 | 6 | 6 |
|  | 4 | 8 | 4 | 6 | 8 | 1 | 2 | 3 | 3 |  | 5 | 5 | 5 | 6 | 1 | 4 | 6 | 8 | 9 |
|  | 6 | 1 | 2 | 0 | 2 | 9 | 2 | 0 | 6 |  | 0 | 5 | 8 | 3 | 2 | 3 | 3 | 1 | 1 |
| R1 | A | A | G | G | A | A | A | C | G |  | T | A | C | A | A | T | A | C | T |
| R2 | . | G | . | . | . | . | G | . | . |  | . | . | . | . | . | . | . | . | C |
| R3 | . | G | . | A | . | . | . | . | . |  | . | . | . | . | . | . | . | . | C |
| R4 | . | . | A | . | . | . | . | . | . |  | . | . | . | . | . | . | . | T | . |
| R5 | . | G | . | A | . | . | . | . | . |  | . | G | . | . | . | . | . | T | . |
| R6 | G | G | . | . | . | G | . | A | C |  | C | . | . | G | G | . | G | T | G |
| R7 | . | G | . | . | . | G | . | A | C |  | C | . | A | G | G | . | G | T | G |
| R8 | G | . | . | . | G | G | . | A | C |  | C | . | . | G | . | . | G | T | G |
| R9 | . | G | . | . | . | G | . | G | C |  | C | . | A | G | G | C | G | . | G |
| R10 | G | . | . | . | . | G | . | A | C |  | C | . | . | G | . | . | G | T | G |
