# Supplementary material for: Evolution of East Asia’s Arcto-Tertiary relict Euptelea (Eupteleaceae) shaped by Late Neogene vicariance and Quaternary climate change
Source: BMC Evol Biol. 2016 Mar 22;16:66. doi: 10.1186/s12862-016-0636-x (PMC4802896; doi:10.1186/s12862-016-0636-x)
Supplement: Additional file 6: Table S5. — Mean estimates of genetic diversity and differentiation at eight nuclear microsatellite loci surveyed across 33 Euptelea populations (marked with an asterisk in Additional file 2: Table S1). (DOC 30 kb) [file 12862_2016_636_MOESM6_ESM.doc]

**Additional file 5: Table S5.** Mean estimates of genetic diversity and differentiation at eight nuclear microsatellite loci surveyed across 33 *Euptelea* populations (marked with an asterisk in Table S1).

| Locus | *N*_A_ | *H*_O_ | *H*_S_ | *H*_T_ | *F*_ST_ |
| --- | --- | --- | --- | --- | --- |
| EP04 | 29 | 0.571 | 0.812 | 0.935 | 0.134 |
| EP06 | 27 | 0.768 | 0.855 | 0.939 | 0.093 |
| EP10 | 11 | 0.232 | 0.257 | 0.564 | 0.552 |
| EP59 | 11 | 0.383 | 0.516 | 0.725 | 0.294 |
| EP87 | 7 | 0.31 | 0.296 | 0.588 | 0.504 |
| EP91 | 15 | 0.479 | 0.603 | 0.832 | 0.281 |
| EP278 | 21 | 0.811 | 0.763 | 0.865 | 0.12 |
| EP294 | 29 | 0.697 | 0.721 | 0.874 | 0.179 |
| Mean | 18.75 | 0.531 | 0.603 | 0.790 | 0.270 |
| Total | 150 | 0.531 | 0.603 | 0.79 | 0.242 |

*N*_A_, observed allele number; *H*_O_, observed within-population heterozygosity; *H*_S_, expected within-population (gene) diversity; *H*_T_, overall gene diversity; *F*_ST_, among-population differentiation.
